# Posterity of nanoscience as lipid nanosystems for Alzheimer's disease regression

**DOI:** 10.1016/j.mtbio.2023.100701

**Published:** 2023-06-17

**Authors:** Shaikh Sheeran Naser, Dibyangshee Singh, Subham Preetam, Shristi Kishore, Lamha Kumar, Aditya Nandi, Faizan Zarreen Simnani, Anmol Choudhury, Adrija Sinha, Yogendra Kumar Mishra, Mrutyunjay Suar, Pritam Kumar Panda, Sumira Malik, Suresh K. Verma

**Affiliations:** a KIIT School of Biotechnology, Kalinga Institute of Industrial Technology (KIIT-DU), Bhubaneswar 751024, Odisha, India; b Institute of Advanced Materials, IAAM, Gammalkilsvägen 18, 59053 Ulrika, Sweden; c Amity Institute of Biotechnology, Amity University Jharkhand, Ranchi, Jharkhand 834001, India; d School of Biology, Indian Institute of Science Education and Research, Thiruvananthapuram, Kerala 695551, India; e Mads Clausen Institute, NanoSYD, University of Southern Denmark, Alison 2, 6400 Sønderborg, Denmark; f Department of Physics and Astronomy, Uppsala University, Box 516, SE-75120 Uppsala, Sweden

**Keywords:** Anti-alzheimer's disease therapy, Nanomedicine, Lipid nano systems, Neurodegenerative diseases

## Abstract

Alzheimer's disease (AD) is a type of dementia that affects a vast number of people around the world, causing a great deal of misery and death. Evidence reveals a relationship between the presence of soluble Aβ peptide aggregates and the severity of dementia in Alzheimer's patients. The BBB (Blood Brain Barrier) is a key problem in Alzheimer's disease because it prevents therapeutics from reaching the desired places. To address the issue, lipid nanosystems have been employed to deliver therapeutic chemicals for anti-AD therapy in a precise and targeted manner. The applicability and clinical significance of lipid nanosystems to deliver therapeutic chemicals (Galantamine, Nicotinamide, Quercetin, Resveratrol, Curcumin, HUPA, Rapamycin, and Ibuprofen) for anti-AD therapy will be discussed in this review. Furthermore, the clinical implications of the aforementioned therapeutic compounds for anti-AD treatment have been examined. Thus, this review will pave the way for researchers to fashion therodiagnostics approaches based on nanomedicine to overcome the problems of delivering therapeutic molecules across the blood brain barrier (BBB).

## Graphical abstract

**Figure:**
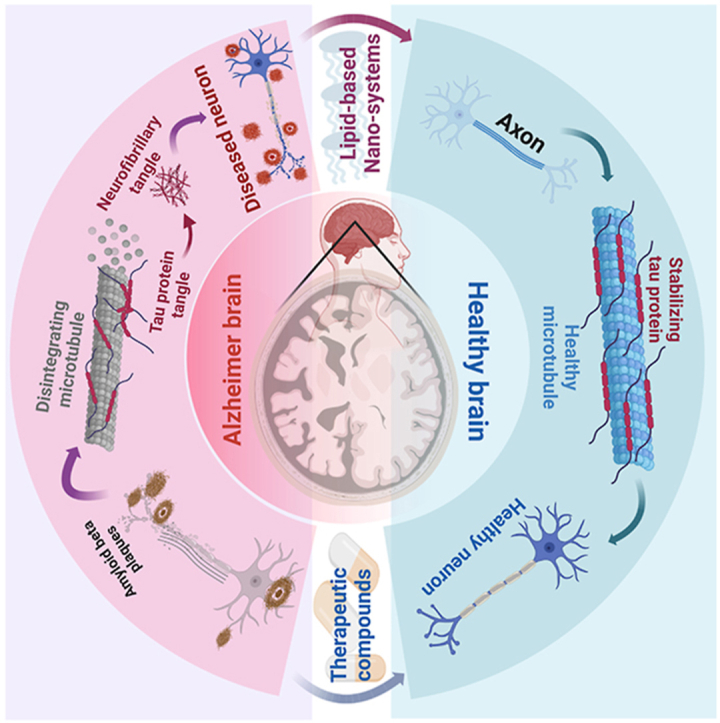

## 1 Introduction

More than 80% of cases of dementia in senior individuals throughout the world are caused by Alzheimer's disease (AD), one of the most prevalent neurodegenerative diseases [1]. It causes a gradual reduction in mental, behavioral, operational, and learning abilities***.*** Late-stage AD patients experience consequences that include trouble adjusting the physical location, needing assistance moving about, significant weight gain or loss, progressive speech loss, and significant problems with short- and long-term memory [2]. Worldwide, there are more than 55 million dementia patients, 60% of whom reside in countries with low or middle incomes. There are around 10 million new cases reported each year. A wide range of brain injuries and diseases can cause dementia. The most prevalent form of dementia, Alzheimer's disease, may be a contributor in 60–70% of cases. AD is caused by the accumulation of protein fragments called beta-amyloid plaques or tau protein tangles [3]. These accumulations prevent neurons from establishing the vital interconnections that are required in memory recollection. Healthy neurons are surrounded by beta-amyloid plaques that prevent them from affixing to one another. Neurons are destroyed and eventually die as a result of beta-amyloid plaques and oligomers, which prevents them from processing synapses [3,4]. Oligomers are commonly assembled much before the beta-amyloid plaques occur. The most hazardous beta-amyloid category has been identified as oligomers [5]. There is currently no known therapy or cure for AD. Drugs are only used to treat symptoms when identified with AD. Positron emission tomography is required for the diagnosis of AD in order to detect the buildup of beta-amyloid protein, one of the proteins that may be responsible for the brain diminution associated with AD [6,7]. It is characterized by developing intracellular neurofibrillary tangles and depositing extracellular amyloid plaques (senile plaques), two pathological markers in the brain [8]. Along with these histopathological markers, several cellular processes, including microglia and astrocyte activation, synaptic dysfunction, axonal transport failure, and even neuronal death, develop as the disease in the brain parenchyma progresses. The second hallmark of AD is the presence of tau proteins, which are located in the axons of nerve cells. Tau proteins' first role is to create microtubules for the movement of essential nutrients throughout the nerve cells. The nerve cells internal nutrients enable the cells to stay robust and straight. When tau tangles form within neurons, their function is compromised [9,10].

There are merely a few symptomatic therapies readily available and there is currently no effective treatment, despite the large number of probable molecular targets identified in the literature and considerable positive evidence from animal models [11,12]. Since AD has a multifactorial and complex etiopathology, there are many reasons why this might be the case, first, the lack of the precision and specificity of anti-AD drugs; second, the incapacity of the majority of drugs to pass through the blood-brain barrier (BBB); third, selection of a single target for evaluating efficacy; and fourth, selecting patients in an advanced state of pathological conditions. Due to the dearth of reliable, early diagnostic markers, patient recruitment in the earliest phases of the condition was originally challenging. Currently, CSF biomarkers, blood biomarkers, MRI, Amyloid PET, Tau PET, and other technologies are used to address this issue [13]. To establish the various phases of pathophysiological progression, characterize systems-based intermediate endophenotypes, and validate multi-modal novel diagnostic systems biomarkers, these combinatory and additional approaches are being used in addition to one another [14]. All of these findings could promote trial designs for clinical interventions that are more resilient [15]. Targeting Tau buildup or hyper-phosphorylation, as well as amyloid formation or accumulation, has been the focus of several techniques. Additionally, a variety of substances, including cholinesterase inhibitors, some kinase inhibitors, glutamate receptor antagonists, calcium or reactive oxygen species modulators, cholinergic transmission modulators, and glutamate receptor antagonists, have been tested in preclinical and clinical trials [11,12]. The few symptomatic therapies are restricted to the targeting of cholinergic deficits and glutamatergic dysfunction, which is unfortunate given that many candidate medications have failed to show a therapeutic effect in established, early, or prodromal illness, or in individuals with a high risk of developing AD [16].

Nanotechnology has gain a considerable interest in last decade for their utility in different biomedical and environmental applications [[17], [18], [19], [20]]. Many new organic and inorganic nanoparticles has been in quest of synthesis by the researchers for these application given the fact of their biocompatibility and the process of their synthesis [[21], [22], [23]]. Nano-encapsulation of therapeutic molecules has several advantages and aids in overcoming constraints such as physical instability and premature bioactive degradation inside the body [[24], [25], [26]]. For the past thirty years, researchers have studied the natural carriers referred to as liposomes for a variety of illnesses, including malignancy [27,28]. Core components utilized to create liposomes include qualities such as biocompatibility, biodegradability, and low toxicity [[29], [30], [31]]. Although the potential for therapy was established in 1961 [32], liposomes have only lately been acknowledged as carriers to enter the central nervous system (CNS). Liposomes are a significant tool for drug delivery and brain targeting because they are biocompatible, highly stable, improve peripheral circulation, and they render it simple to bind ligands to their surfaces for receptor-mediated drug administration. The drugs or therapeutics agents can be delivered to the brain more efficiently and safely by these nanoparticles, improving their pharmacokinetic and pharmacodynamics attributes [33,34]. Through the application of drug delivery methods based on nanotechnology, the curative efficacy of the medicines can be improved [35,36]. The regulated release of pharmaceuticals at a specific spot is the primary advantage of nanomedicines for the medical treatment of neurological disorders like AD [37]. To improve drug loading, they are made by integrating a combination of liquid and solid lipids, which are then stabilized using emulsifiers or aqueous surfactant solutions [36,38]. As a multidisciplinary field, the engineering of these NPs for drug delivery offers novel perspectives and opens windows for the manipulation of substances with a minimum of one dimension sized from 1 to 150 ​nm [[39], [40], [41]]. Numerous sectors, such as medicine, pharmacy, chemical/biological detection, and optics have reaped advantages from the advancements achieved by nanomedicine. The focus of this work is to provide a centralized location for current information concerning the potential of lipid-based nanosystems as a drug delivery tool for the therapeutic management of Alzheimer's disease. The complications associated with drug transport to the brain are also addressed in brief. This review discusses recent studies on lipid-based nanoparticles, including their potential as a therapy for Alzheimer's disease, and illustrates some of the methods by which lipid-based nanosystems can be modified and adapted.

## 2 Molecular pathogenesis of AD

**Fig. 1:**
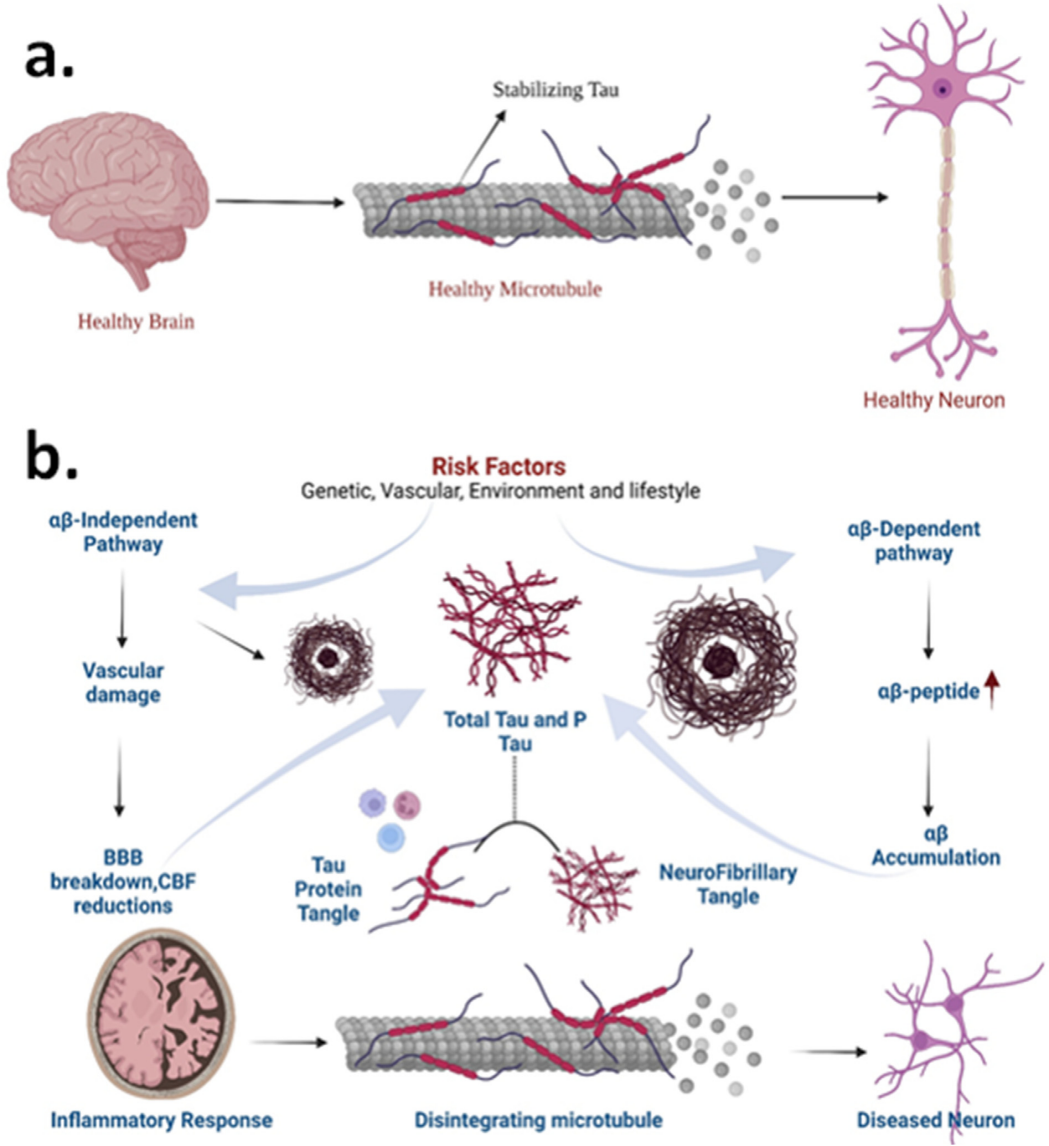
Molecular patho-mechanism of a) Healty brain b) Alzheimer's disease.

Several pathogenic situations are thought to hasten the course of AD. These variables cause severe loss of the sections of the brain, like the hippocampus and cortex, in the early phases of AD [42]. The pathophysiology of Alzheimer's disease has been studied using many hypotheses, such as the NMDA excitotoxicity theory, the tau hypothesis, the amyloid hypothesis, and the cholinergic hypothesis [43]. The disease is characterized by cholinergic pathway degradation and beta-amyloid (Aβ) buildup. The level of the neurotransmitter acetylcholine (ACh) is shown to be reduced in the cholinergic hypothesis, which is mainly owing to enhanced activity of the enzyme acetylcholinesterase (AChE) and cholinergic neurodegeneration [44]. ACh levels are lowered in the hippocampal and cortical areas, which are crucial in the function of memory [45]. Anti-cholinesterase medicines, such as Galantamine, Rivastigmine, and Donepezil, are used in pharmacotherapy to address this hypothesis. Butyrylcholinesterase (BuChE), in addition to AChE, is being studied to help with cognitive impairment. Rivastigmine is a medication that inhibits both the enzymes, BuChE and AChE [46]. The amyloid hypothesis suggests clumps of the Aβ peptides of up to 40–42 activate mediators of inflammation such as JNK and TLR4, causing neurons to die [44]. The enzymes that degrade APP and- and β-secretases produce soluble toxic clumps known as Aβ fragments [45]. These Aβ fragments are considered the primary source of neurodegeneration in Alzheimer's disease shown in Fig. 1. Under healthy conditions, the α-secretase works on APP to create fragments of APPα that are soluble (sAPPα). These pieces linger in the extracellular environment. The 83-amino acid carboxy-terminal (C83) elements, which anchor to the cell membrane, are also generated [[47], [48], [49]]. The sAPPα is responsible for improving the plasticity of synapses, memory, learning, and memory by modulating the excitability of neurons and increasing the resilience of neurons to oxidative and metabolic stressors [49]. APP is cleaved by β-secretase-1 (BACE1) into sAPPβ in the extracellular environment and a fraction bound to the membrane with 99 ​amino acids (C99) during a neuropathological condition. The C99 fragment is subsequently processed by γ-secretase, producing either Aβ (1−42) or Aβ (1−40) peptides. These Aβ peptides are thought to be involved in forming senile plaques. Although sAPPα is advantageous, Aβ peptides have been linked to changes in the metabolism of energy, disruption in the homeostasis of calcium, dysfunction of mitochondria, oxidative stress, reduced neural plasticity, and synaptic loss [49]. One of the main goals of AD treatment is to eliminate or prevent the production of these fragments of Aβ, as well as to improve the patient's survival, life quality, and function. Inhibition of the aggregation of Aβ, modification of the production of Aβ, immunotherapy focused against Aβ, and an increase in the degradation of Aβ are among the many therapeutics that target Aβ being studied [50]. Many treatments targeting Aβ peptides proved unsuccessful in clinical trials, and efforts to solve the problem are still underway. Several genes linked to AD cause neurodegeneration by enhancing the amounts of Aβ peptides in the brain. Tau protein helps to keep microtubules stable, especially in axons [51]. Tau hyperphosphorylation leads to the creation of neurofibrillary tangles (NFTs). NFTs have paired filaments that are helical and insoluble that induce neurodegeneration. The hallmarks of Alzheimer's disease are tau proteins that are hyperphosphorylated and plaques of Aβ. Only if the disease's curative therapy stops or reverses the patient's symptoms will it be referred to as such. The modest and continuous stimulation of receptors of NMDA causes chronic “excitotoxicity,” which progresses to neurodegeneration. Even minor depolarization of the cell membrane can disrupt Mg^2+^ blockage of the receptor channels of NMDA, resulting in a pathogenic input of Ca^2+^ into the postsynaptic neurons [52]. The loss of synaptic function, accompanied by synapto-toxicity and the cell's death, occurs due to this Ca^2+^ excess over time. Drugs like Memantine, which are given later in AD to counteract the excite-toxicity of NMDA, only provide relief for the symptoms [[53], [54], [55]].

## 3 Challenges posed by the blood-brain barrier

**Fig. 2:**
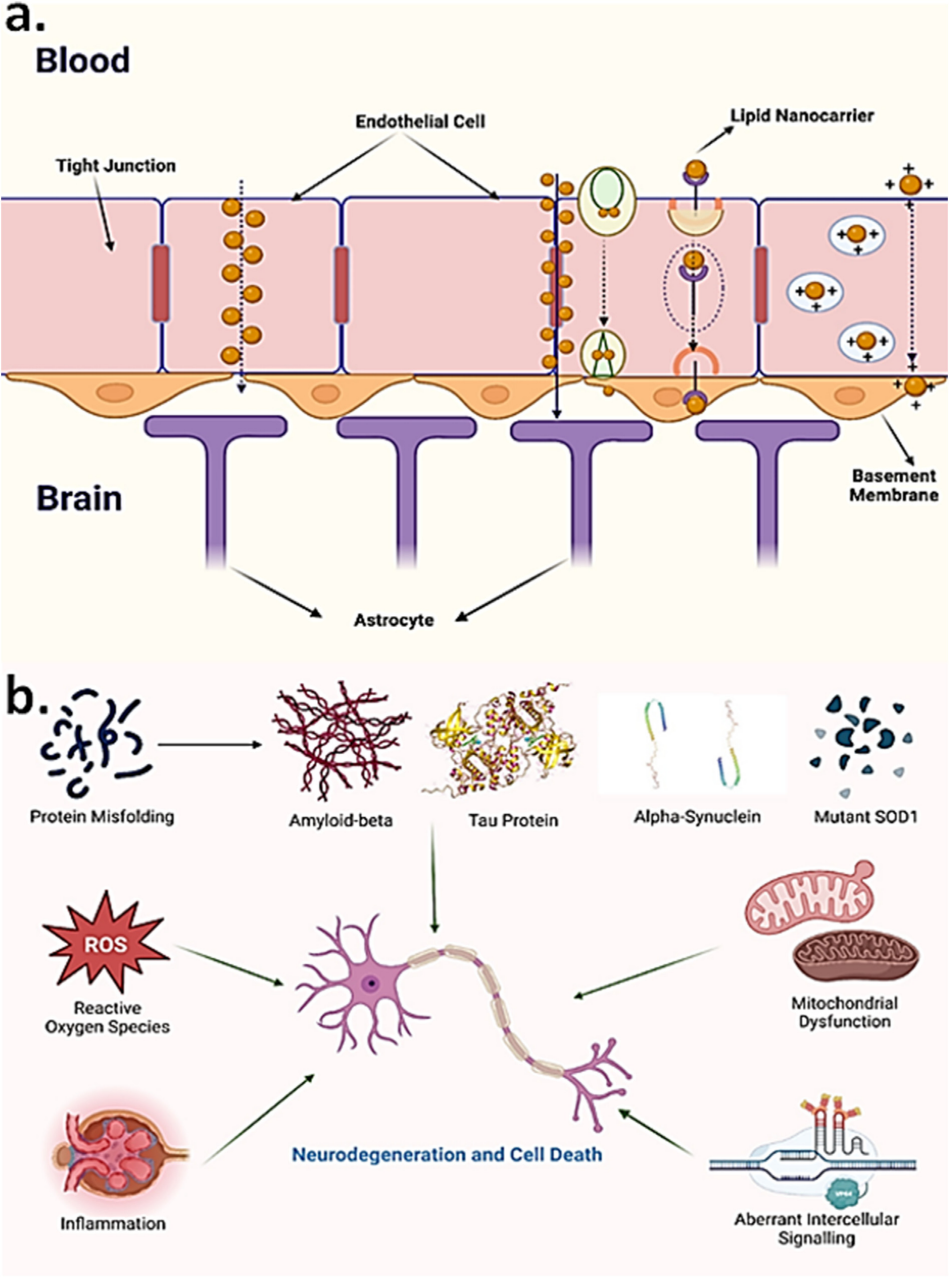
Challenges posed in delivery of drug molecules and therapeutic compounds for AD regression by (a) Mechanistic representation of lipid nano-carriers crossing blood brain barrier [65,66] (b) Factors responsible for neurodegeneration and cell death [66].

The CNS has a variety of barriers, such as the blood–spinal cord barrier, blood-retinal barrier, BBB, and blood-cerebrospinal fluid (CSF) barrier, to protect the CNS from infectious agents, harmful chemicals, and numerous types of cells, such as circulating blood cells, neurotoxic molecules, and pathogens that could be invading it [56,57]. The BBB is a carefully regulated barrier that exists between the CNS and the bloodstream and is made up of pericytes and neural endothelial cells that are scattered along the membrane. The BBB is a significant barrier to overcome in the treatment of AD. This is due to factors such as tight control between the CNS and the circulation and efflux pumps. The presence of adherens junctions (AJs) and tight junctions (TJs) within the cerebral endothelial cells ensures the BBB's extremely constrained control [58]. TJs, also known as zonulae, occlude, are a significant characteristic of the BBB and consist of numerous proteins, including structural proteins such as junctional adhesion molecules (JAMs), occludin claudins, and regulatory proteins ZO-3, ZO-1, ZO-2, and cingulin, among others. The expression of these proteins and their unique arrangement and interactions required for BBB control [59] are shown in Fig. 2. Almost all neurons in the human brain have vasculature, with capillary cellular volume accounting for only 0.2 to 0.1% of total brain volume [60]. Microvessels comprise 95% (roughly) of the BBB's total area and are the main route for medication delivery. Small holes in the CNS capillaries hinder solutes from traveling quickly to the brain via the bloodstream. A plethora of enzymes with metabolic functions serves as a metabolic barrier in BBB endothelial cells, altering both endogenous and external molecules. Ectoenzymes on the plasma membranes of capillary endothelium, pericytes, and astrocytes include aminopeptidases, endopeptidases, and cholinesterase [61]. Several efflux transport mechanisms have been discovered and characterized at the BBB, and these systems are critical for protecting the brain from possible poisons [62]. Thus, many essential drugs struggle to reach the brain in the concentration needed for effective treatment. Depending on the size and features of the molecules, several methods for transporting them over the BBB exist. The paracellular transfer of ions and polar solutes between the brain's extracellular fluid and the endothelium's cells from the blood's plasmid is usually minimized by their concentration gradients [63]. However, the BBB contains Solute carriers (SLC) that act as transporters for essential building components like carbohydrates in the form of glucose and amino acids. Apart from that, transcellular transport and ATP-binding cassette transporters (ABCs) are involved in the movement of lipophilic substances and are being exploited for therapeutic drug delivery [64]. The BBB restricts ions and polar solutes, particles larger than 1000 ​Da, and various small compounds, including lipophilic medicines. As a result, crossing the BBB is a critical step in the treatment of PD. As a result, it can be deduced that these multiple transit routes through the BBB are crucial for better understanding and developing drug delivery techniques for target molecules.

## 4 Characterization and synthesis of lipid-based nano-systems

### 4.1 Liposomes

**Fig. 3:**
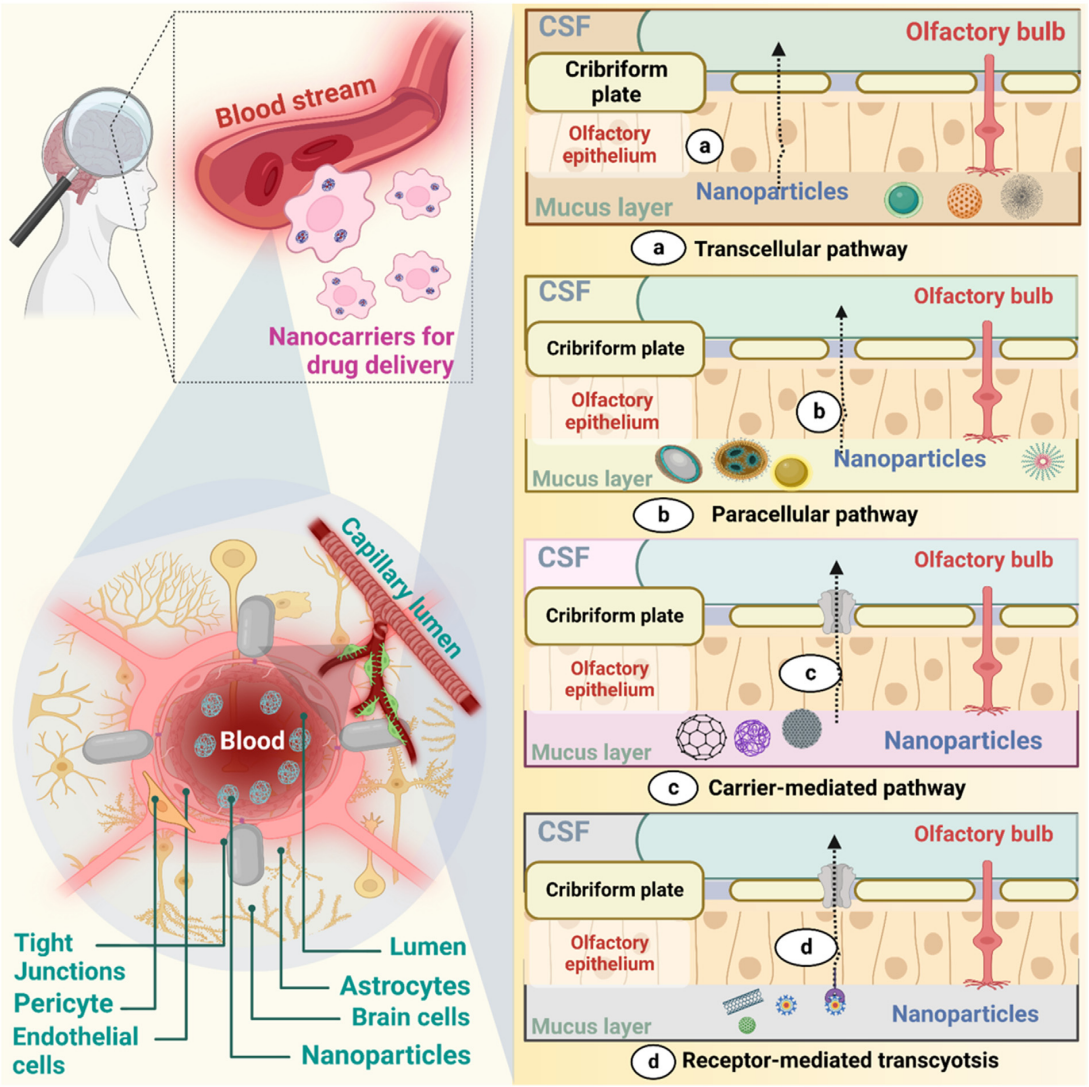
Graphical representation of different transport mechanisms of NLC across BBB via receptor-mediated transport, carrier-mediated drug transport, Para cellular drug diffusion, and transcellular drug transport.

**Fig. 4:**
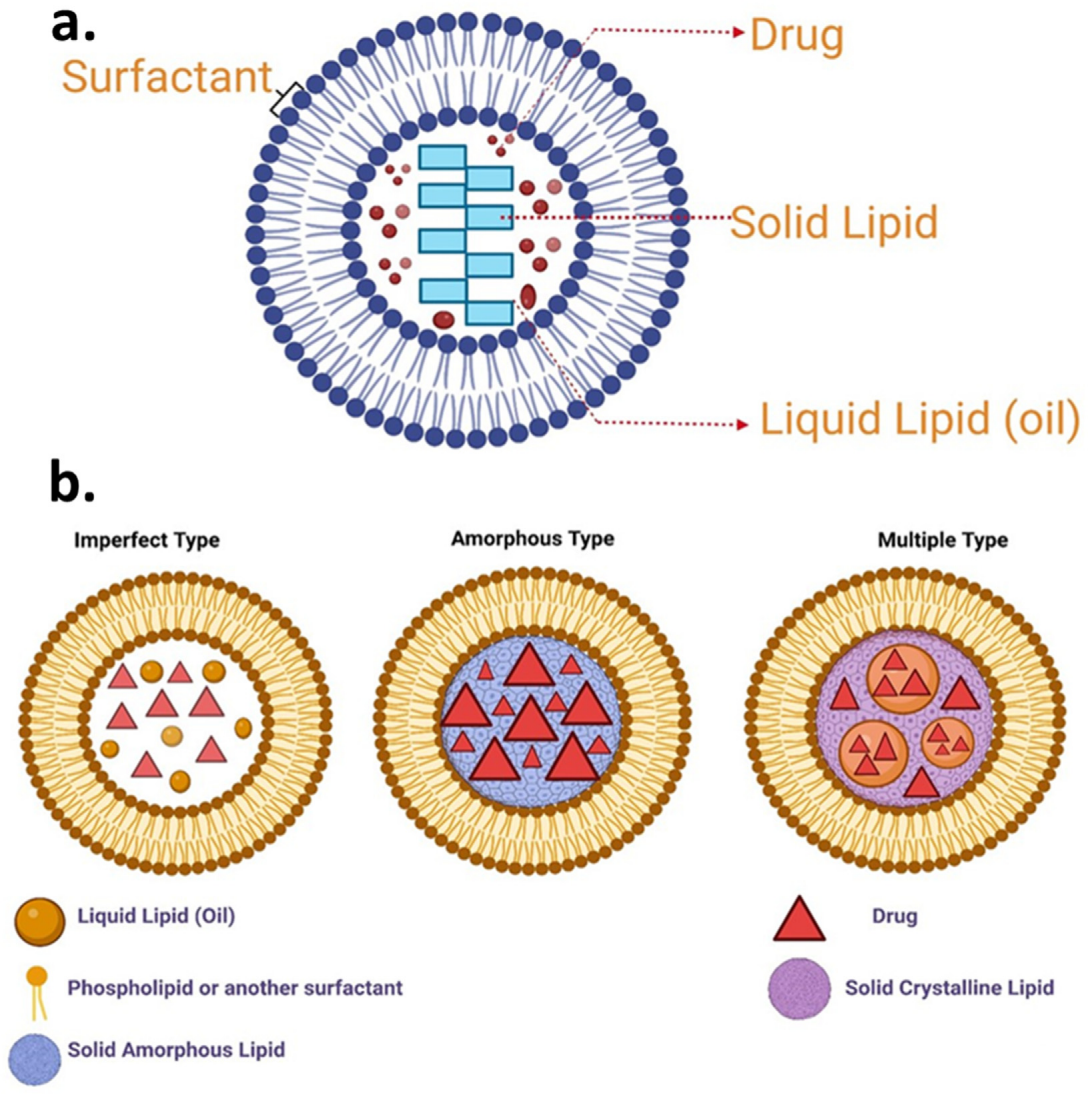
(a) Structure of NLC drug delivery system [70] (b) Schematic diagram showing different types of NLC [71,72].

Liposomes are a relatively new suspension nanomaterial, having been demonstrated as a system for a therapeutic agent in the 1970s [67]. These are spherical shape particles with a hydrophilic compartment in the central portion surrounded by one or more lipid bilayers, as a core aspect of the cell membrane delivery system for drugs, providing them with structural similarity to peptides and proteins [68]. Based on their dimensions and bi-layer volume, they are classified as tiny single lamella with a size around 10–50 ​nm, sizeable single lamella around 50–1000 ​nm in diameter, and several lamellar around 20–100 ​nm in diameter. Multilamellar vesicles (MLVs, >0.5 ​m, >5 bilayers) are formed when multiple bilayers entrap aqueous fluid (Fig. 3). These structures are resolvable due to interactions of non-covalent such as London dispersion forces and electrostatic dipole-dipole interaction between molecules. Liposome formation must raise the heat above the lipid phase transition temperature. The multiple processing techniques, fat type (neutral, uncharged, and charged), lipid composition, surface-active agent, organic solvent, and ionic strength of the suspension medium used in the strategies all have an impact on the liposome's properties (particularly size, size distribution, and lamellarity) (Fig. 4). A pH jumping approach made SUVs (20–60 ​nm) from MLVs. Apart from MLVs, GUVs approximately 10 ​m in diameter can be easily made by slowly hydrating lipid films over a considerable amount of time (1–48 ​h) without trying to shake. Low concentration levels (10 ​mM NaCl) are GUVs from gentle hydration adequate. Traditional film hydration technologies are limited in their application due to their shared ability entrapment, non-scalability, and size variability. Microfluidic hydrodynamic focusing (MHF) was developed for the rapid synthesis of lipid membranes in a microchannel environment [68,69] (Table 1).

**Table 1:** Characterization of lipid-based nanosystems used to deliver therapeutic compounds for AD regression.

Nano system	Characteristic	Method of synthesis	Interaction	Reference
Liposomes	zeta potential is −43mV, the surface charge is 70–350 ​nm	PH jumping approach, Microfluidic hydrodynamic focusing	electrostatic, hydrogen bond, Van der Waals, steric repulsion	[73]
Nano emulsions	surface charge is nearly zero, and the zeta potential of 30 ​mV.	High energy methods-homogenization and ultrasonication, Low-energy methods-low, interfacial tension	repulsive electrostatic interactions, steric hindrance, hydrophobic interactions	[74]
NLCs	Its zeta potential is−31 ​mV to −36 ​mV, and its surface charge is 40–1000 ​nm	Amalgamation	Electrostatic repulsion	[75]
SLNs	Its surface charge is 50 ​nm, and its zeta potential is 11–61 ​mV	High-pressure homogenization (HPH), solvent emulsification/evaporation, supercritical fluid extraction of emulsions (SFEE), ultrasonication or high-speed homogenization, and spray drying	electrostatic interaction	[76]
Polymeric Nanoparticles	surface charge is 1–1000 ​nm, −18.4 ​mV zeta potential	sequential synthesis of core and shell structure and self-assembly of block copolymers.	Electrostatic interaction	[77]
Nanocrystals	N/A	HNC	Vander wall force	[69]

### 4.2 Nano-emulsions

The dispersed phase (droplets) is distributed throughout the continuous phase and has a colloidal interfacial boundary with various sizes of 100–500 ​nm [78]. There are two methods for making nano-emulsions: low-energy and high-energy methods. High-energy procedures such as dynamic HPH and ultrasonication use a lot of energy (108 ​W/kg) to split large droplets down to around 100 ​nm in size. Because of their brute force strategy, high-energy approaches can reliably produce nano-emulsions with dispersed phase volume concentrations as high as 40%. Low-energy techniques use low interfacial tension to reduce droplet size with energy input from a magnetic stirrer. This allows for a simple and scalable path to nano-emulsion creation without further shearing [74]. They are used as the administration of drugs mode has recently been acknowledged and encouraged to address several issues with traditional delivery systems, including limited bioavailability, poor target recognising ability, and BBB penetrability. The primary drivers of nano-emulsions adaptabilities are various oils and surface modifiers utilized in preparation. Nano-emulsions constituted from omega-3 polyunsaturated fatty acids (PUFA) oils, impart specific properties to nanocarriers that help them overcome biological barriers, like BBB, and thus aid in the rapid delivery of drugs to peripheral sites, particularly the brain [79]. A nano-emulsion technology based on pine-nut oil significantly increased paclitaxel oral bioavailability.

### 4.3 Nanostructured lipid carriers (NLCs) and solid-lipid nanoparticles (SLNs)

**Fig. 5:**
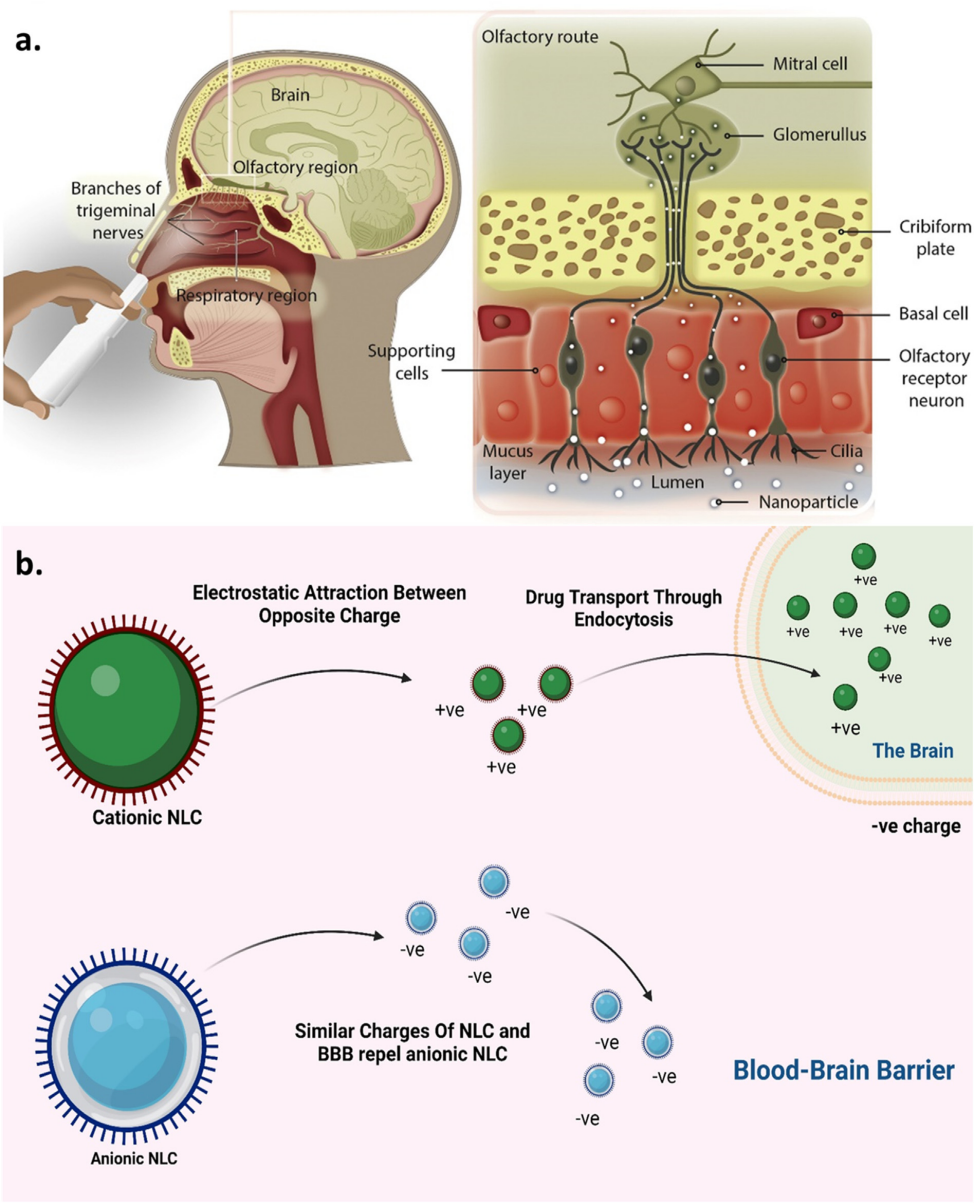
(a) Schematic representation of the administration of drug-loaded NLC and the transport mechanism of drug-loaded NLC through olfactory and trigeminal nerves directly to the brain [83]. Figure showing the structure of olfactory nasal mucosa and neuronal network in the nasal epithelium, which assists the drug transport either by Para cellular route or from the transcellular mechanism. (b) Effect of surface charge on the permeability of NLC across BBB. The positively charged NLC gets attracted by the negatively charged BBB and hence can easily cross the barrier and enters into the brain. In contrast, the similarly trusted BBB repels the negatively charged NLC.

SLNs and NLCs are gaining some traction as novel drug carriers because they are at the frontline of the fast-changing nano-delivery system [80]. These nanocarrier systems that are aqueous and colloidal are made up of physiological lipids (waxes, steroids, fatty acids, and triglycerides) dissolved in solutions of an aqueous surfactant or water and can solidify once cools down. Due to the advantages such as regulated release, stability, protection of labile pharmaceuticals from degradation, physical strength, and low intrinsic cytotoxicity, SLNs are preferred over PNPs for drug delivery in the brain [80]. One of the approaches performed to improve the drug's loading capacity and the long-term stability of SLNs was the production of NLCs via the fusion of spatially distinct lipids or the merging of liquid lipids with solid lipids (Fig. 5). To address the potential issues with SLNs, NLCs were created as the next generation of SLNs at the end of the 1990s. NLCs aid in drug expulsion prevention, loading capacity, and storage stability. They are not to be confused with SLNs. By the constituents of the solid matrix. Solid and liquid lipids coexist in the lipid phase of NLCs at room and room temperatures [81]. Sterylamine-based SLNs usually contain the antipsychotic drug clozapine, have been established and shown to effectively deliver drugs into intravenous and intraduodenal administration in the brain Quercetin-loaded SLNs are another drug-loaded SLN that are used to treat AD [82]. In a rat model of amyotrophic lateral sclerosis (ALS) induced by immunization with the experimental allergic encephalomyelitis, riluzole-loaded SLNs were proven more efficient than free one's riluzole.

LNPs can be made using various methods, such as solvent emulsification/evaporation, spray drying, high-speed homogenization, ultrasonication or high-pressure homogenization (HPH), and supercritical fluid extraction of emulsions (SFEE). The HPH devised two methods, hot and cold, in which the medication is dispersed or solubilized in a lipid heated to 5–10° Celsius beyond its melting point [84]. Solid fat ranging from 0.1 to 30% by weight is dispersed in an aqueous phase in SLNs. To improve reliability, surfactants are used in amounts ranging from 0.5 to 5%. Dimension of particles, storage stability, loading of the drug, and release behavior patterns can all be influenced by the fats and surfactants used. At both body and ambient temperatures, their lipid components are solid.

**Fig. 6:**
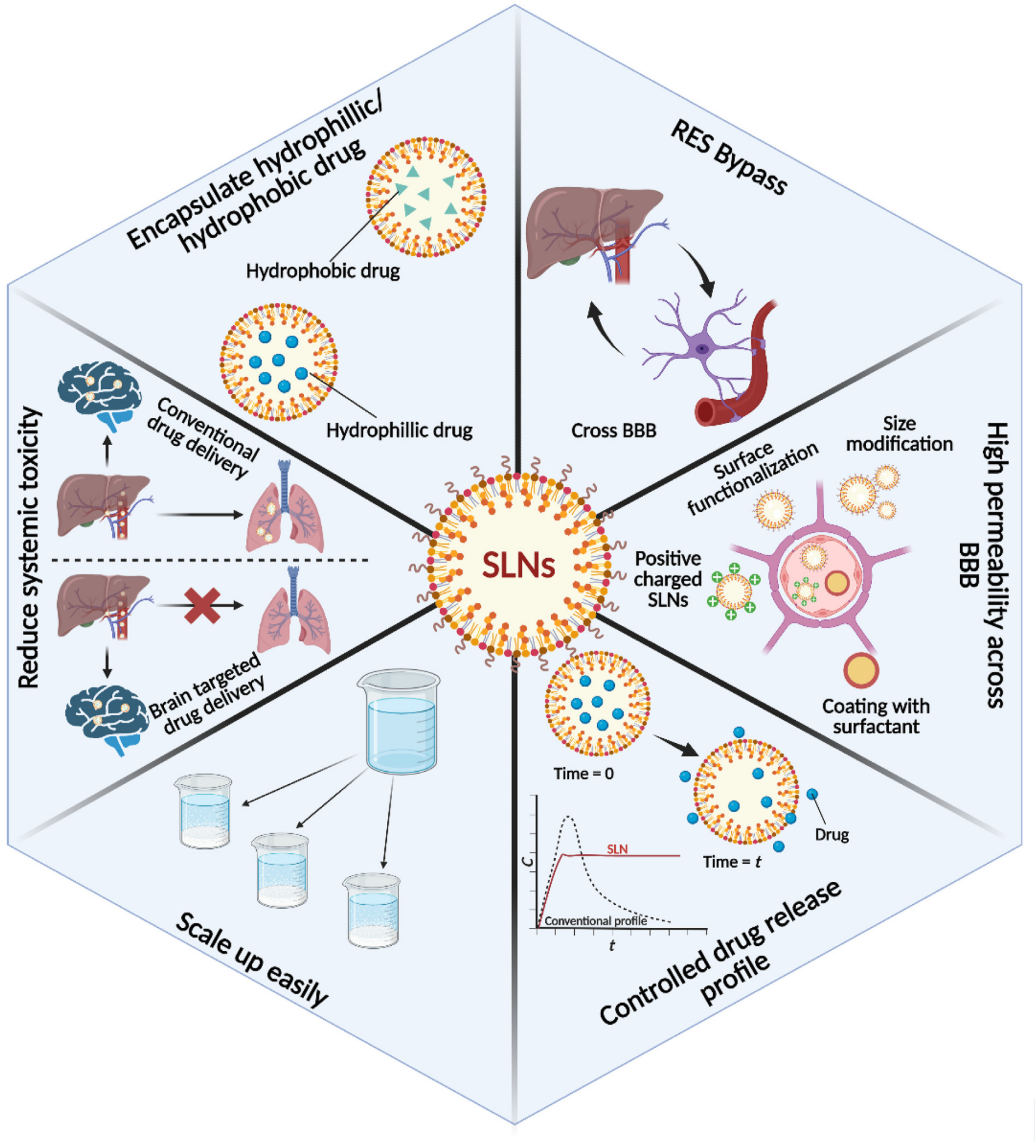
Advantages of employing SLNs in AD drug delivery and treatment.

**Fig. 7:**
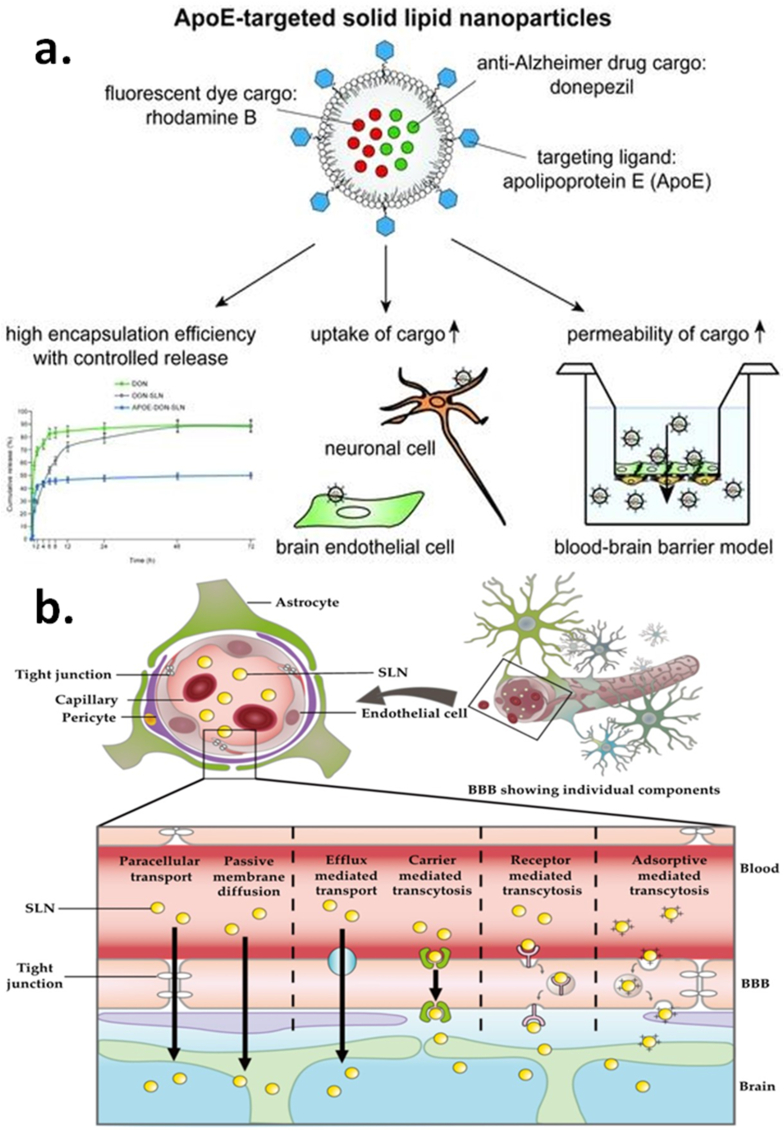
SLNs for AD drug delivery (a) SLN-loaded drugs transport mechanism via BBB, (b) Apolipoprotein-targeted SLNs for CNS targeting [88].

SLNs comprise palmitic acids, corticosteroids, waxes, acylglycerols, diacylglycerols, and neutral fat, among other lipids. SLNs can be used to make both hydrophobic and hydrophilic drugs based on the synthesis method. SLNs have several benefits over the other systems, such as ease of processing, low price, large-scale production, excellent physical consistency, great release profile, chemical versatility, preparation without organic solvent, hardly a toxic effect of the lipid delivery system, lipid biodegradability, being less expensive than polymeric carriers, and durability [85,86]. At room temperature, NLCs are modified SLNs containing solid and liquid lipids in their lipidic phase (Fig. 6). SLNs are NLCs that have been altered to have a mixture of solid and liquid phases, resulting in an amorphous matrix that tends to increase stabilization and load capacity. SLNs and NLCs have various properties that make them suitable for drug delivery via parenteral, cutaneous, pulmonary, and topical routes (Fig. 7). Such combinations were used to reduce the potentially harmful side effects of the potent pharmaceuticals and increase the treatment's efficacy. They've also shown promising results in gene transfer, cosmetics, and the food industry [87].

## 5 Characterization of therapeutic compounds used for anti-AD treatment

A number of therapeutic compounds has been recognised as anti-AD treatment. Some of them has got special attention by physicians because of their high efficacy and biocompatibility (Table 2)**.** A few with detailed mechanism has been listed and defined.

**Table 2:** Characterization of therapeutic compounds used for an anti-AD activity.

Drug/Therapeutic compound	Preclinical	Anti-AD hallmarks activities	Clinical significance	References
HUPA	*In vivo*	It can protect neurons against Aβ-induced oxidative injury and apoptosis, ameliorate mitochondrial malfunction, promote non-amyloidogenic APP processing, regulate NGF, reduce iron in the brain, and antagonize NMDA-R.	It has non-cholinergic effects on AD.	[89]
Galantamine	*In-vivo/In vitro*	This allosterically modulates nicotinic acetylcholine receptors (nAChRs) and inhibits acetylcholinesterase (AChE).	It regards conventional cholinesterase inhibitors.	[90]
Nicotinamide	*In vivo*	In 3xTg-AD mice, a competitive inhibitor of sirtuins or class III NAD ​+ ​-dependent HDACs. Nicotinamide, like SirT1, preferentially decreases a particular phospho-species of tau (Thr231) involved with microtubule depolymerization.	It works on tau phosphorylation at Thr231 and may regulate tau stability.	[91]
Quercetin	*In vivo*	Dietary quercetin supplementation may reverse the effects of Aβ on these pathways. Further investigations in the brain employing in vivo RNAi of the cell cycle protein cyclin B improved AD phenotypes, confirming that cell cycle-related proteins mediated quercetin's therapeutic effects in AD.	Quercetin has been reported to inhibit Aβ toxicityin vitro ​and ​in vivo.	[92]
Resveratrol	*In vitro*	The extracellular buildup of soluble A oligomers was found to be substantially responsible for AD dementia and memory problems in Tg2576 mice in recent research. Treatment with polyphenolic chemicals derived from grape seed extract (GPSE) reduced A peptide oligomerization and cognitive decline in amyloid Tg2576 mice. It also improves amyloid beta-peptide clearance and decreases neuronal damage.	Resveratrol helps promote non-amyloidogenic cleavage of the amyloid precursor protein.	[93]
curcumin	*In vivo*	Curcumin is lipophilic, meaning it may pass through all cell membranes and exert its effects intracellularly. Curcumin inhibits the proliferation of microglia. Curcumin has an impact on neuroglial proliferation and differentiation at low doses. It stops microglia from proliferating and differentiating. In THP-1 monocytic cells, curcumin reduces A-induced Egr-1 protein production and Egr-1 DNA-binding activity. The involvement of Egr-1 in amyloid peptide-induced cytochemokine gene expression in monocytes has been studied.	Curcumin's many benefits, such as decreased Beta-amyloid plaques, delayed neuron degeneration, metal chelation, anti-inflammatory, antioxidant, and decreased microglia production, have improved overall memory in Alzheimer's patients.	[94]

### 5.1 HUPA

**Fig. 8:**
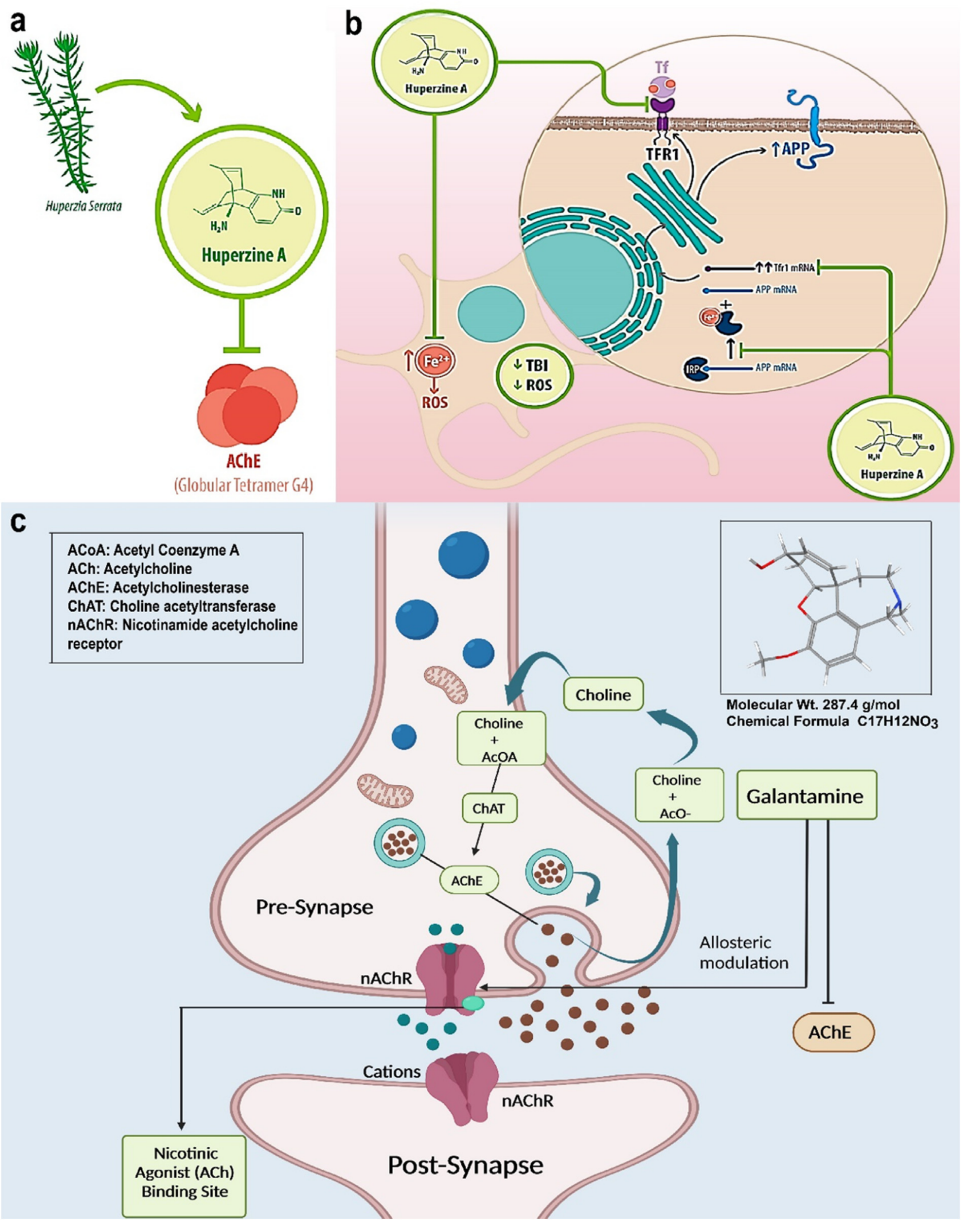
Mechanisms of action of (a),(b) HUPA-A [99] (c) Galantamine therapeutics used for anti-AD treatment.

Huperzine A is a cholinesterase inhibitor, a medication that works by boosting neurotransmitter levels in the brain [95]. Huperzine A has been shown to improve memory and protect nerve cells, potentially slowing cognitive decline in Alzheimer's patients. Huperzine A may help people with Alzheimer's disease improve their mental performance. For the treatment of Alzheimer's disease, HUPA (huperzine A) is loaded into lipid nanosystems. The cognitive loss brought on by amyloid-beta plaques in the brain is a hallmark of Alzheimer's disease [95]. HUPA is an acetylcholinesterase inhibitor that reduces symptoms by boosting the availability of acetylcholine (Fig. 8). HUPA has problems with solubility and clearance, though. These problems are resolved by HUPA being enclosed in lipid nanosystems, which also provide stability and biocompatibility. They efficiently deliver HUPA to the brain and can be delivered in several ways [96]. The qualities of HUPA are enhanced by this tailored delivery, which also lessens unwanted effects while boosting therapeutic results. There is hope for Alzheimer's patients as research is being done to improve lipid nanosystems and increase medicine delivery effectiveness [97,98].

### 5.2 Galantamine

Galantamine is a medication that treats mild to moderate AD and other memory-based problems [100]. It is a synthetically manufactured alkaloid found in the bulbs and blooms of *Galanthus nivalis* and *Galanthus caucasicus*. Like other cholinesterase inhibitors, Galantamineight not work for mild cognitive impairment [101]. Galantamine causes the inhibition of acetylcholinesterase, an enzyme that hydrolyzes acetylcholine, consequently increasing acetylcholine's availability for synaptic transmission [100]. Galantamine binds to nicotinic receptors' allosteric regions, causing them to change conformation (Fig. 8c). The nicotinic receptor's responsiveness to acetylcholine is improved by this allosteric regulation [101]. Acetylcholine availability is increased by activating presynaptic nicotinic receptors, which increases acetylcholine release. Thus, in a nutshell, galantamine's dual mechanism of action includes acetylcholinesterase competitive inhibition and allosteric nicotinic modulation (Table 2).

Galantamine is incorporated into a lipid matrix using lipid film hydration or solvent evaporation methods. The resulting lipid nanosystems can encapsulate galantamine within their lipid bilayers or disperse it in their aqueous core. They can reach the brain through systemic circulation or direct administration into the cerebrospinal fluid, facilitated by their small size and biocompatibility. Once in the brain, the lipid nanosystems release galantamine in a controlled manner, maintaining therapeutic drug levels and minimizing side effects. This targeted and sustained delivery promises to improve treatment outcomes for Alzheimer's disease [100,102,103].

### 5.3 Resveratrol

**Fig. 9:**
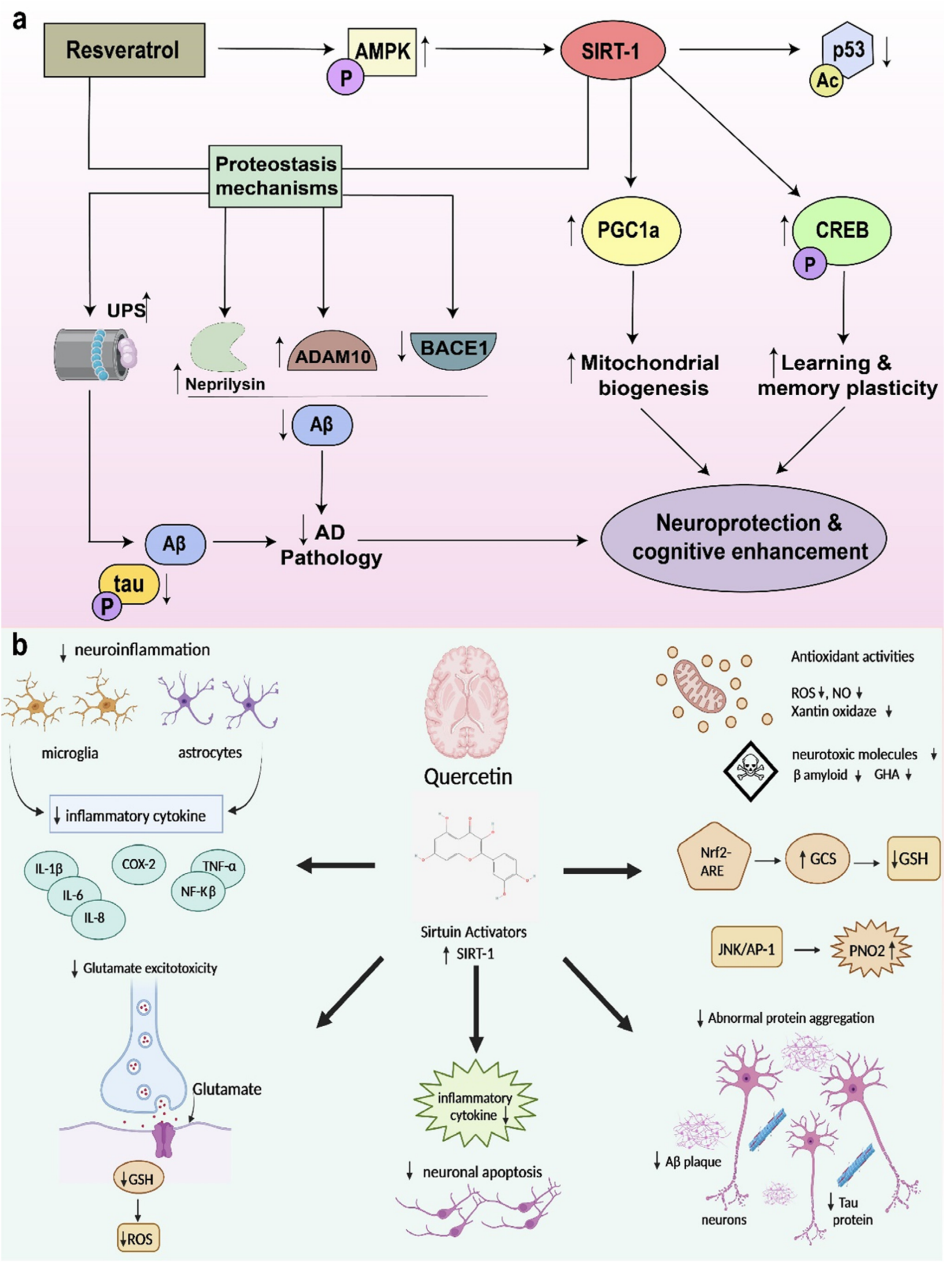
Mechanisms of action of therapeutics used for anti-AD treatment (a) Resveratrol (b) Quercetin.

Resveratrol is a phenolic compound that protects against pathogens such as bacteria and fungi and is present in various plants, particularly grape skins and seeds [104]. It shows a link between moderate red wine consumption and a low risk of cardiovascular disease, a phenomenon called the “French Paradox” [105]. Resveratrol has a broad range of biological effects, including anticarcinogenic properties, cardioprotective, vaso-relaxing, antioxidant, phytoestrogenic, and anti-inflammatory (Fig. 9a), all of which aid in the diagnosis of cardiovascular diseases and cancers, and also degenerative brain disorders like Alzheimer's disease [93]. For the delivery of therapeutic medicines, such as Resveratrol, in Alzheimer's disease, lipid nanosystems present a viable strategy. However, due to its weak solubility and bioavailability, resveratrol's potential as an Alzheimer's treatment is constrained. Resveratrol can effectively pass the blood-brain barrier by being enclosed within lipid nanosystems, which allow for its delivery to the brain [106]. Lipid nanoparticles' tiny size permits targeted distribution and improved brain penetration. Further improving selectivity and minimizing off-target effects is functionalizing lipid nanosystems with targeted ligands. Through the improvement of Resveratrol's stability, solubility, and targeted distribution, this strategy shows significant promise for boosting treatment outcomes for Alzheimer's. For clinical applications and optimization, more study is required [107,108].

### 5.4 Quercetin

Alzheimer's disease is characterized by Aβ build-up in the brain parenchyma and aberrant hyperphosphorylation of the intermediate filament protein tau inside neurons, which results in neurofibrillary tangles. The existence of these inclusions causes synaptic loss, especially in the neocortex and hippocampus, oxidative stress, and neuroinflammation, eventually leading to neuronal malfunction and death (Fig. 9b). Tau protein and human islet amyloid polypeptide deposits have also been found in the pancreatic islets of T2DM patients [109]. Quercetin is a polyhydroxy flavonoid in food plants such as vegetables and fruits' flowers, leaves, and fruits. Many Chinese herbal treatments contain quercetin, including *cortex moutan 5, Hypericum perforatum, Hypericum perforatum, Ginkgo biloba leaves,* and *Sophora japonica*. The bioactivities of quercetin, such as antioxidant, anti-inflammatory, hypoglycemic, and hypolipidemic properties, are essential to the pathogenesis of Alzheimer's disease [110]. Quercetin, a naturally occurring substance with anti-inflammatory and antioxidant effects, is being loaded onto lipid nanosystems to treat Alzheimer's disease. Cognitive loss brought on by the buildup of amyloid-beta plaques and neurofibrillary tangles in the brain. Although quercetin may have therapeutic benefits, its low bioavailability and limited capacity to pass the blood-brain barrier pose difficulties [111]. Lipid nanosystems provide a remedy by encasing quercetin and enhancing its stability and targeting capabilities. These nanoparticles can be used orally or intravenously, effectively delivering quercetin to the brain. This targeted delivery strategy seeks to raise quercetin levels in the brain, which could improve therapeutic results and have fewer side effects [111]. To improve drug delivery and expand Alzheimer's treatment options, current research focuses on optimizing lipid nanosystems [111,112].

### 5.5 Curcumin

**Fig. 10:**
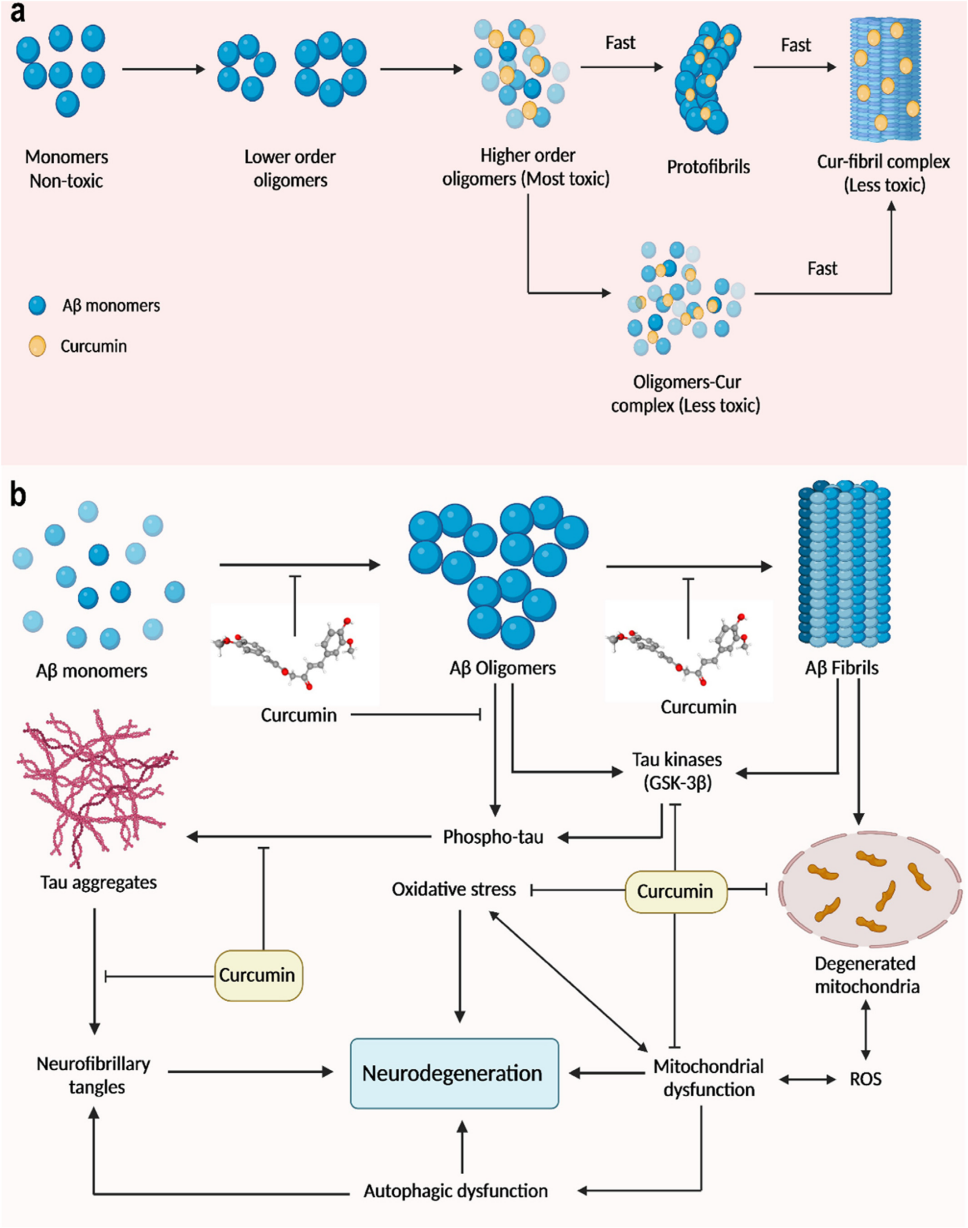
(a) Curcumin fibril complex form from monomeric subunits of curcumin and Aβ protein (b) Mechanisms of action of curcumin used for the anti-AD treatment.

Since it has a high affinity for amyloid and fluorescence that is natural, curcumin has been used as an early probe for diagnosis. Due to its multi-target effects, curcumin protects and prevents many chronic diseases, including hyperlipidemia, hypertension, and cerebrovascular disease. Curcumin has been found to efficiently preserve the typical structure and function of synapses, mitochondria, and cerebral arteries in treating and preventing AD, decreasing risk factors for several chronic diseases, and lowering the risk of AD [113]. Curcumin's anti-amyloid and metal iron-chelating properties and anti-inflammatory and antioxidation properties affect AD through multiple signalling pathways (Fig. 10). Curcumin's rational use in the prevention and treatment of AD. Curcumin binds to Aβ plaques and emits a strong fluorescence signal, making it a useful diagnostic tool for AD. Curcumin is one significant possibility for delivery of treatments using lipid nanosystems in Alzheimer's disease. Despite having limited therapeutic application, curcumin contains anti-inflammatory and neuroprotective effects [114]. By encasing curcumin, lipid nanosystems like liposomes and nanoparticles address this. These methods have benefits like stable delivery, improved solubility, effective blood-brain barrier penetration, and the capacity to target plaques. When the nanosystems reach the brain, they release curcumin under controlled conditions, lowering inflammation, preventing plaque formation, and enhancing neuronal survival. Lipid nanosystems have the potential to improve the therapeutic effects of curcumin in Alzheimer's disease [94,115].

### 5.6 Nicotinamide

**Fig. 11:**
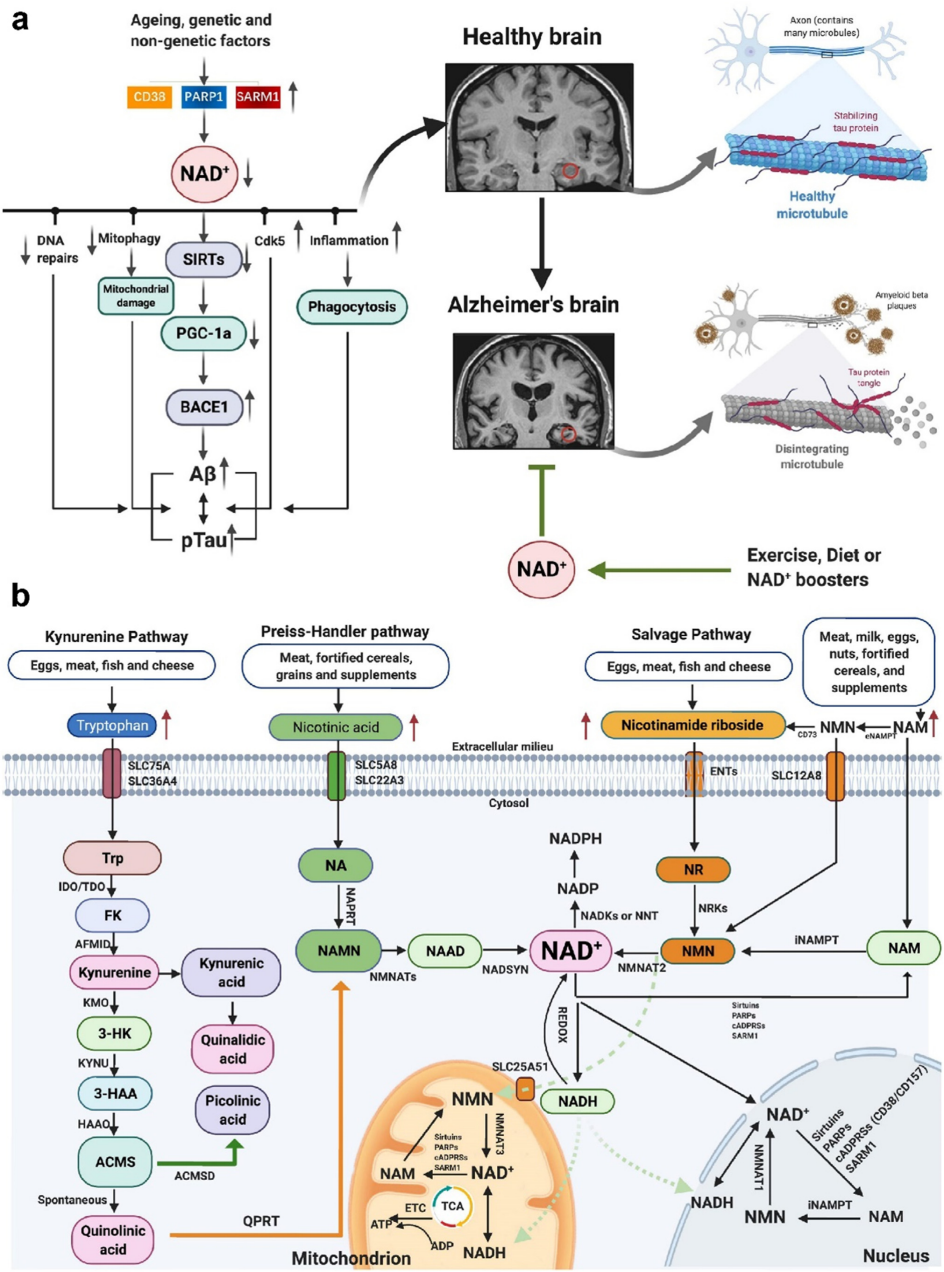
(a) Role of factor influencing AD b) Mechanisms of action of nicotinamide used for anti-AD treatment. (Adapted and modified from Ref. [118]).

Nicotinamide is a type of vitamin B3 that may be used to treat AD owing to its neuroprotective properties. Nicotinamide inhibits the brain sirtuin deacetylase and increases the amounts of microtubule-stability-associated proteins (Fig. 11). In 3xTg-AD mice, nicotinamide decreases a particular phosphotau species (Thr231) while raising p25 levels, restoring cognitive impairments associated with AD pathology [116]. Thr231-phosphotau is one of two tau species known to inhibit microtubule polymerization and is a common CSF biomarker for AD [109]. Liposomes have emerged for delivering therapeutic agents in the treatment of Alzheimer's disease. One specific application is the loading of nicotinamide, a form of vitamin B3 with neuroprotective properties. Nicotinamide can help mitigate the pathological processes associated with Alzheimer's, but its delivery to the brain is challenging. Encapsulating nicotinamide within the lipid matrix protects it from degradation and allows targeted delivery to the brain. Surface modifications enable active targeting, reducing off-target effects Lipid nanosystems offer a solution by encapsulating and protecting nicotinamide, enhancing its solubility and enabling efficient transport across the blood-brain barrier. These nanosystems can be designed with surface modifications for targeted delivery and incorporation of other agents, opening possibilities for combination therapies and treatment monitoring [117].

### 5.7 Rapamycin

**Fig. 12:**
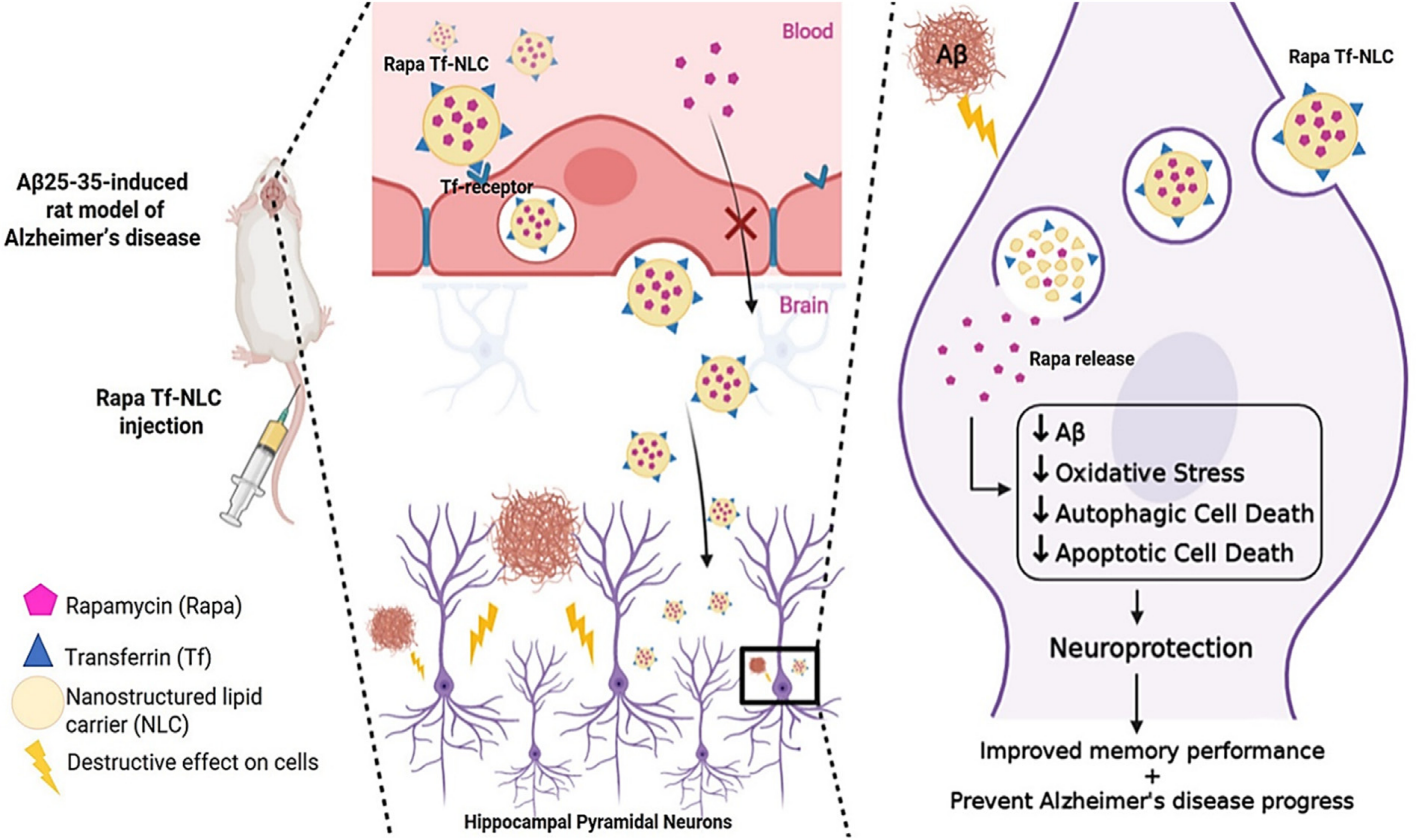
Application of NLCs in treating AD Transferrin-decorated NLCs as rapamycin delivery system in Alzheimer's disease.

Rapamycin is an effective treatment for neurodegenerative disorders because it blocks the action of the mammalian target of rapamycin (mTOR) protein. To promote Aβ and phosphorylated TAU protein degradation, Rapamycin can induce cell autophagy (Fig. 12). The decrease in the production of Aβ and phosphorylated TAU protein can be regulated by mTOR as it regulates the level of β-secretase, γ-secretase, and cAMP-dependent protein kinases which are responsible for Aβ and phosphorylated TAU protein secretion [119,120]. It can reduce the level of inflammation and ROS in AD lesions while also protecting nerve cell survival and plasticity [121]. A study conducted by Lei et al., used a nanocleaner, designed with Rapamycin core and surface modified with KLVFF and acid-cleavable DAG peptide. The ROS responsive PLGA core and surface modified peptides make [R@(ox-PLGA)-KcD] nanoparticle that can be used for targeted delivery and collaborative treatment. The result showed that the nanoparticle could specifically capture Aβ while also mediating the disordered lesion microenvironment for additional treatment [119].

### 5.8 Ibuprofen

Ibuprofen is a common non-steroidal anti-inflammatory drug (NSAID) used in case of Alzheimer's disease that might influence the AD pathology. A study conducted by Lim et al., used transgenic mouse models of AD and administered ibuprofen orally. The result showed that the anti-inflammatory drug ibuprofen was successful in reducing the amyloid deposition upon early stage administration [122]. The insufficient permeability of BBB and intra-brain distribution of drugs makes it difficult for the treatment of AD. Another study conducted by He et al., established a co-delivery system Ibu&FK@RNPs using ibuprofen and FK506 for encapsulation that can target the advanced glycation end products receptor (RAGE) and respond to ROS production in AD [122]. The result demonstrated that the co-delivery system reduced neurological damage and neuroinflammation in mice model used for AD. Such targeted drug delivery strategies can be used for the treatment of AD in future [122].

## 6 Theragnostic potential of lipid-based nano-systems for anti-AD therapy

### 6.1 Drug delivery

**Fig. 13:**
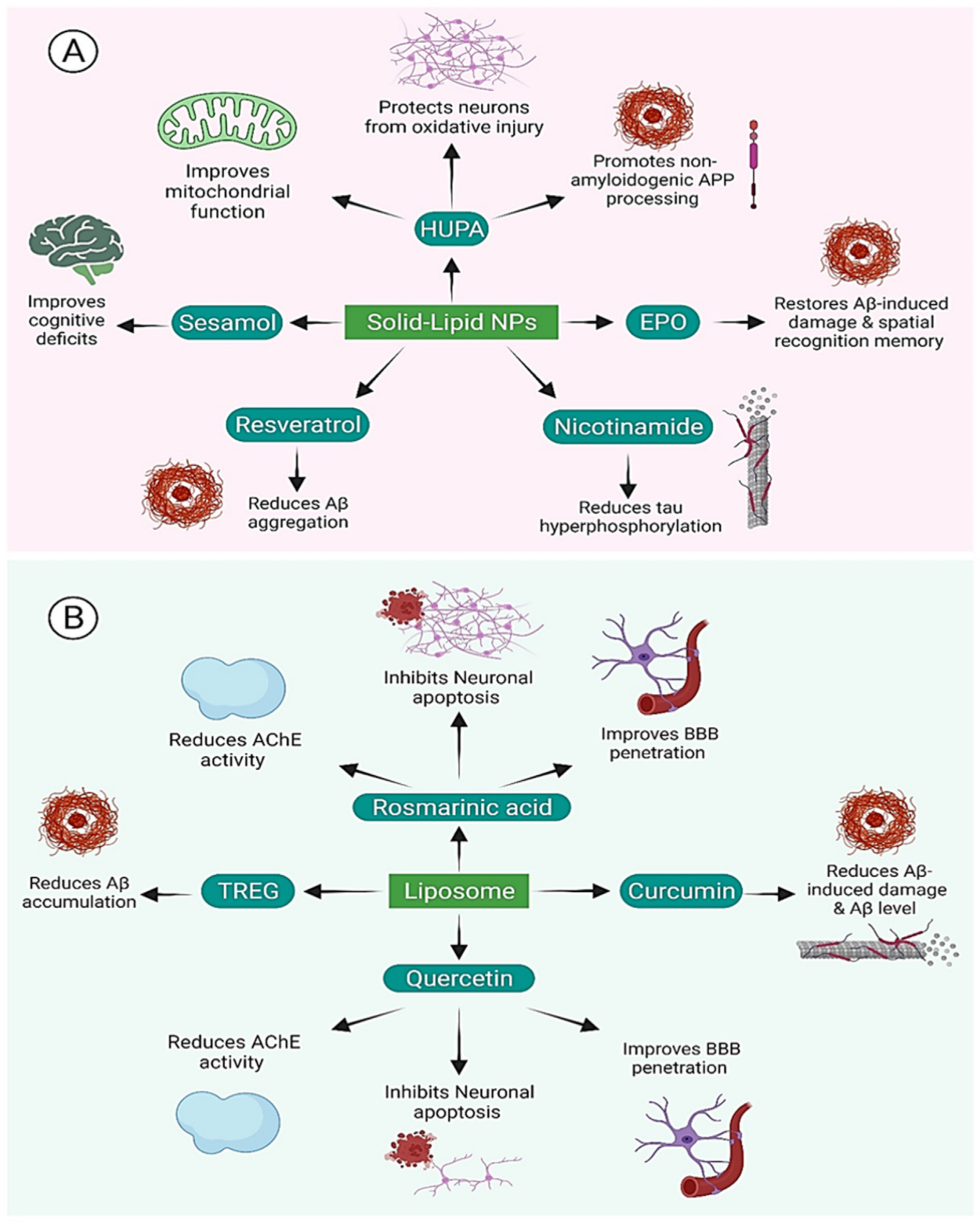
Therapeutic role of lipid based nanosystems for anti-AD activity. (a) Application of solid-lipid-based nanosystems to deliver therapeutic compounds to immunomodulate the AD hallmarks. (b) Application of liposome for drug delivery causing AD regression.

The primary problem posed by AD is the delivery of drug molecules and therapeutic compounds. So, concerning drug delivery, the primary objective of solid lipid nanoparticles (SLN) is to increase bioactivity and efficacy while also controlling non-specific toxicity, immunogenicity, pharmacokinetics, and pharmacodynamics. SLN poses several benefits, such as ease of mass production, biodegradable and biocompatible substances, the limited possibility for toxic effects, the power to control and alter drug release, enhancement of drug solubility, to deliver therapeutics with low cytotoxicity, and immunogenicity, high nucleic acid encapsulation efficiency, and potentiation. Herein we have discussed the clinical significance of the application of lipid nanosystems for drug delivery and summarized in Table 3 and illustrated in Fig. 13.

**Table 3:** Delivery of drug molecules and therapeutic compounds for AD regression utilizing lipid nanocarriers.

Lipid Nanoparticles	Functional Ligand	Pre-clinical	Drug Delivery	Characteristic	References
Solid Lipid Nanoparticles	N/A	*In-Vitro*	HUPA	It protects neurons from oxidative injury and apoptosis caused by A, improve mitochondrial dysfunction, antagonize NMDA-R, regulate NGF, promote non-amyloidogenic APP processing, and reduce iron in the brain.	[123]
Solid Lipid Nanoparticles	N/A	*In-Vivo*	EPO	In comparison to rats treated with free EPO, EPO-SLN protected the rat's brain from A-induced damage, and spatial recognition memory was significantly restored. The reduction of oxidative stress and the ADP/ATP ratio, as well as the prevention of A plaque deposition.	[124]
Solid Lipid Nanoparticles	N/A	*In-Vivo*	Sesamol	Rats' cognitive deficits were restored. Nitro-oxidative stress and cytokine release are reduced.	[125]
Solid Lipid Nanoparticles	N/A	*In-Vivo*	Galantamine	In comparison to free drugs, there was a 2-fold increase in bioavailability and improvement in cognitive impairment in rats.	[126]
Solid Lipid Nanoparticles	OX26 antibody	*N/A*	Resveratrol/grape seed extract	Transcytosis is higher in functionalized SLN than in unfunctionalized SLN. Aggregation of the A peptide is reduced.	[127]
Solid Lipid Nanoparticles	Polysorbate 80, phosphatidylserine, and phosphatic acid	*In-Vivo*	Nicotinamide	In rats, the treatment improved memory preserved more neuronal cells and reduced tau hyperphosphorylation when compared to the control group.	[116]
Liposomes	Tfr, penetratin	*In-Vitro*	ApoE encoding pDNA	ApoE expression has increased. When compared to nontargeted and single-modified Tfr or penetratin preparations, brain targeting and gene delivery efficiency were improved.	[128]
Liposomes	N/A	*In-Vitro*	Microbial keratinases Ker1 and Ker2	In vitro digestion capacity for Abeta fibrils.	[129]
Liposomes	ApoE, phosphatidic acid and polysorbate 80	*In-Vivo*	Rosmarinic acid, quercetin	Rosmarinic acid and quercetin have improved BBB penetration and neuronal targeting. Neuronal apoptosis is inhibited, and cell viability is increased. AChE activity is reduced, and lipid peroxidation is reduced. A plaque formation is reduced.	[130]
Liposomes	Cardiolipin, phosphatidic acid and TAT peptide	*In-Vivo*	Nerve growth factor, rosmarinic acid, curcumin and quercetin	Downregulation of several protein kinases involved in the physiopathology of Alzheimer's disease Therapeutics' ability to bypass the BBB has improved. In the Wistar rat brain, increased viability of SK-N-MC cells damaged by A fibrils and prevention of hyperphosphorylated tau production.	[131]
Liposomes	ApoE, *anti*-Tfr	*In-Vitro*	TREG	In vitro model with improved transport across the BBB. Ability to bind to Aβ and prevent it from aggregating. In double transgenic mice, the brain/liver ​+ ​spleen ratios are higher.	[132]
Liposomes	N/A	*In-Vivo*	Rivastigmine hydrogen tartare	Liposomes had a 4-fold increase in plasma and brain drug levels when compared to rivastigmine intranasal solution.	[133]
Liposomes	N/A	*In-Vivo*	Donepezil	In rats, donepezil has a higher brain uptake than the free form.	[127]
Liposomes	Tfr	*In-Vivo*	Alpha-M	BBB crossing is more efficient. In comparison to -alpha-M liposomes and free alpha-M solution, higher bioavailability and -M concentration in the rats' brains.	[134]
NLC	Lf	*In-Vivo*	Curcumin	Greater uptake of BCECs, higher accumulation in disease-affected areas of the rat brain, and superior efficacy in preventing damage	[115]
NLC	N/A	*In-Vivo*	Rivastigmine	Rats treated with rivastigmine-loaded NLC in situ gel had better memory function than rats treated with resveratrol suspension given orally.	[133]
MEs	N/A	*In-Vivo*	Metformin, Borneol	In comparison to Met-W/O/W MEs and Met free drug systems, B-Met-W/O/W MEs had a longer residence time, longer half-life, slower release rate, and higher drug targeting index in the brain, demonstrating that borneol improved and enhanced metformin distribution to the CNS.	[135]
MEs	N/A	*In-Vivo*	Huperzine	Improved cognitive performance.	[96]
MEs	N/A	*In-Vivo/In-Vitro*	Piperine	Drug efficacy and delivery to the brain have both improved. In rats induced with sporadic dementia of Alzheimer's type, the free drug had a better therapeutic outcome. Superior anti-apoptosis and anti-inflammatory activity in the brain	[136]
MEs	N/A	*In-Vivo*	Thymoquinone	In rats fed a high fat/cholesterol diet, thymoquinone NEs improved memory, reduced An aggregation, and provided neuroprotection.	[137]
NEs	N/A	*In-vitro*	Naringenin	When compared to free naringenin, it has a better in vitro neuroprotective effect against A neurotoxicity. In SH-SY5Y cells exposed to A, there were lower levels of hyperphosphorylated tau and less amyloidogenesis.	[125]

Yang et al. used an *in-vitro* solid-lipid nanoparticle system to deliver HUPA to patients with Alzheimer's disease. The NLC size used to provide HUPA was 120 ​nm, and the surface charge was −22.93 mv. The control drug exhibited from in vitro profile was 89.18% with good loading efficiency, while loading capacity was 1.96%. Thus, it could be concluded that utilizing a solid-lipid nanosystem could be efficient for the clinical patient [131]. In a similar investigation, Patel et al. used in vivo solid-lipid nanoparticles to deliver HUPA (Huperzine-A) for anti-Alzheimer treatment in the clinical patient via MEMs, SLNs, and NLCs for *trans*-dermal delivery in a murine model. After 48 ​h of testing, no skin irritation substance. It is later considered for side use for clinical patients [138].

Cardiolipin (CL), phosphatidic acid (PA), and Liposomes (Lip) with TAT (Transactivation of Transcription) peptide modifications were fashioned and used by Yung-Chih et al. to enhance the delivery of anti-apoptotic particles in the hippocampus and cure AD in clinical patients. TAT is used to penetrate the BBB, while cardiolipin and phosphatidic acid target Tau protein hyperphosphorylation in the brains of wister rats. Hence, TAT peptides are confirmed to diagnose AD with cJun Tau at serine 202, protein kinase, p38, and caspase-3 [131]. PEGylated lip-encapsulating donepezil was developed by Al Asmari et al. for the treatment of AD. In a study of Wistar rats, drastically enhanced brain bioavailability compared to an unlimited drug formulation [127].

Misra et al. used solid lipid nanoparticles, a system in which the particle size would be less than 100 ​nm with entrapment of drug around 83.42 ​± ​0.63% to deliver galantamine for anti-Alzheimer treatment in the patient. The *in-vivo* experiment exhibited 90% more drug volume at a time interval of 24 ​h and thus a better memory-restoration outcome cognitive loss rat compared to naive drug. Hence, galantamine-loaded solid lipid nanoparticles are used as a drug for the treatment of AD [139].

Joana et al. used solid lipid nanoparticles to deliver resveratrol conjugated with grape skin for enhanced efficiency and to overcome A-beta aggregation for anti-AD treatment in clinical patients. Resveratrol exhibited 86% of A-beta peptide at 40 μm concentrations, while grape skin and seed extract produced 92% and 97%, respectively. Solid lipid nanoparticle was mixed with antibody OX26 ​mAB to cross the BBB easily. Endothelial cells show more relevant results than OX26 SLNs in cellular grasp, so resveratrol was taken as a treatment for AD [140].

Rajput et al. used a resveratrol *in-situ* gel to treat Alzheimer's disease. It was made using the melt emulsifying probe sonication method, with particle diameter 13211.90 ​nm, PDI of 0.2090.005, zeta potential −233.79 mv, drug content of 9.263.79%, and entrapment efficiency of 7411.40%, and drug loading of 9.263.79%. Compared to the oral route of resveratrol revocation, the optimized in situ gel has a 5-fold more penetration throughout the nasal mucosa, and awareness in rats administered rivastigmine-loaded NLC gel was enhanced. As a result, resveratrol nanomaterials lipid carried in situ gel is used to treat Alzheimer's patients [124]. Met-loaded MEs held steady by borneol (B-Met-W/O/W MEs) were developed by Hong et al. These fat-soluble medicinal herbs drug can boost the bioavailability of drugs and concentration in the nervous system. When comparison to Met-W/O/W MEs as well as Met free drug systems in pharmacokinetic studies in mice, B-Met-W/O/W MEs had a longer retention time, extended half-life, and slower release rate, and increased drug targeting index in the brain, suggesting that borneol managed to improve as well as increased metformin percentage to the CNS·B-Met-W/O/W MEs are thus used to treat Alzheimer's disease [125]. Despite the best attempts of the pharmaceutical industry, no efficient disease-modifying medication is currently available. Human clinical trials are required for the drugs and delivery methods, and their efficiency in vivo is still to be studied. Various drug delivery methods are being studied, and there is a need to discover and form several more.

### 6.2 Delivery of therapeutic compounds

The primary problem posed in AD is the delivery of therapeutic compounds to the targeted region. The therapeutic compounds need to be transported across the BBB in order to be efficient the anti-AD treatment. Dhawan et al. developed SLNs of quercetin, a promising antioxidant, to target the CNS in the treatment of AD [141]. Using mixtures of Compritol and Tween 80, accomplishing particle shape, and the required drug entrapment, colloidal stability, and release of drug features in the formulation was a huge challenge due to the diverse nature of these polymers. The optimized formulation improved behavioral memory retention in rodents with aluminum-induced dementia significantly. The persistence of nitrate levels, glutathione, and lipid peroxidation inside the brain homogenates of these rodents also revealed quercetin-loaded SLNs' CNS targeting. Because of the considerable reversal of aluminum-induced neurotoxicity accomplished with quercetin-loaded SLNs, SLNs have a lot of potential as a platform technology for attempting to target other natural and synthetic chemicals in the brain to increase efficacy in a range of CNS illnesses. Hence, it is considered a successful experiment using quercetin as a treatment for AD [141].

Yusuf et al. used a polysorbate 80-coated in vivo brain-targeted piperine solid lipid nanoparticle for anti-AD therapy. Piperine in solid lipid nano-formulation of equivalent decreased SOD, increased acetylcholinesterase, and reduced immobility causing reduced plaques and tangles resulting in thus outperforming Donepezil. Hence, it could be inferred that by reducing oxidative stress and cholinergic degradation, P-80-PIP-SLN has shown therapeutic effects in AD [142].

Bruna et al. used ApoE2 to treat AD in mice using transferrin and penetratin-tagged liposomal NPs. APoE2 plasmid was infected through liposomes modified with transferrin-Penetratin in both *in-vitro* and *in-vivo*. Hence, dual ligand-based liposomal gene delivery by targeting the brain as well as gene delivery efficiencies. Furthermore, plasmid ApoE2 could be used to treat AD using transferrin-penetratin modified liposomes [130].

Yung-chih et al. used in vitro liposomes filled with antioxidants to penetrate BBB and impede beta-amyloid to cause neurodegeneration. Both rosemary acid and quercetin-3-*O*-rutinoside are powerful antioxidants that reduce lipid peroxidation and the production of ROS like peroxynitrite leftists, which have been linked to a rise in amyloid fibrils inside the brain [126]. ApoE, not only has the potential to promote brain absorption through binding to numerous targets inside the BBB, but it can also enhance the A binding capacity of linked lipid nanocarriers when combined with PA. Thus, in a nutshell, both in vitro and in vivo experiments exhibited that ApoE-QU-PA-liposomes, ApoE-QU-RA-PA liposomes, and ApoE-RA-PA-liposomes for example, are dual-functionalized nanomaterials possess a significant impact, such as increased lipid transmission and increased lipid transport. Liposomes containing ApoE-QU-RA-PA could thus be used to treat AD [126].

For anti-AD therapy, Konstantina et al. designed multifunctional liposomes with Treg, ApoE, and transferrin receptor antibodies. HcMEC/D3 levels are increased by Treg Lip, and also impede Aβ aggregation through mf-lLIPS via targeting two BBB targeting ligands in *ex-vivo* and *in-vivo* transgenic mice. Hence, liposomes are considered a treatment for anti-AD therapy. To improve the stability of the nanosystem [134]. Nageeb et al. fashioned electrostatic stealth liposomes containing (RHT). The purpose of providing steric and electrostatic hindrance, (DDAB) and PEG-DSPE were introduced. *In vivo, ex vivo,* and *in-vivo* studies, liposomes when compared to RHT intranasal solution, demonstrates a response in plasma and brain drug concentrations. ESS Liposomes (478 ​± ​4.94 ​nm) having zeta potential (ZP-8±0.2mv) were made *in-vivo ex-vivo* and *in-vitro* on both plasma and brain drugs with an efficiency of 48 ​± ​6.22. Hence RHT conjugated liposomes could be used for the treatment of AD [133].

α-Mangostin encapsulated in transferrin-modified liposomes was developed by Zhi-Lan Chen et al. α-mangostin is a xanthone that provides protection and extends the lifespan of cerebral cortical neurons Toxicity of an oligomer. In comparison to α-M liposomes and free α-M solution, Tfr-liposomes enhanced xanthone penetration through the BBB, as a result, bioavailability and α-M intensity inside the brain are increased. Of rat and thus could potentially be utilized for anti-AD therapy [143].

Sachdeva et al. developed sesamol-loaded-SLN to treat Alzheimer's disease. Sesamol obtained from Sesamum species oil has been shown to protect against age-related neurological diseases. In a dose-dependent manner, the SLN was discovered in an AD experimental model in vivo that could successfully re-establish memory impairment in rats given intracerebroventricular streptozotocin, as well as alleviate oxidative stress parameters like nitro-oxidative stress and cytokine release. According to these findings, because SLN seemed to be an efficient system for delivering sesamol to the CNS, it could be investigated as a brain targeting technique for AD [128].

Dara et al. compared the efficacy of EPO-loaded SLN to native erythropoietin inside a rodent model of AD. When compared to rodents given free EPO, EPOSLN was able to protect the brain of rats from the effects of an infusion in the hippocampal region, Furthermore, the prevention of Aβ plaque deposition, as well as the reduction of oxidative stress and the ADP/ATP ratio, were more effective. As a result, solid lipid nanoparticles containing erythropoietin are used to treat AD [144].

Using (S80) from PS, or phosphatidic acid as nanocarriers, Vakilinezhad et al. encapsulated SLN, a hydrophilic drug (PA). The protection of the prepared formulation was evidenced in vitro toxicological studies in the SH-SY5Y cell line, except for S80-functionalized nanocarriers. When compared to non-functionalized nanoparticles, the biodistribution of synthesized SLN in the brain was considerably higher, especially in the first collective, where *P*S-SLN managed to reach larger levels than PA-SLN. In an AD rat model, nanosystems, particularly the *P*S-SLN, were much more successful in the stable delivery and distribution of nicotinamide to the brain than conventional oral nicotinamide, leading to improved neuronal counts in hippocampus subdomains being preserved, and tau hyperphosphorylation is reduced. As a result, PS function solid lipid nanoparticles produce the best nicotinamide results.

Meng et al. developed a new lactoferrin-conjugated NLC that binds curcumin to brain cells and slows the progression of AD. In this research, the use of the identical lipids that end up making up decent density proteins was novel, leading to the creation of an LDL-mimic nanocarrier. The BBB's endothelial cells both have Lf and LDL receptors, which could make it easier to deliver drugs to the brain. *In vitro* studies in BCECs demonstrated that Lf-mNLC has a 1.5-fold up than simple NLC. *Ex vivo* imaging studies revealed that Lf-mNLC has a 2.78-fold higher uptake than plain NLC. As a result, Lf appears to be a real promoter of BBB crossing. As a result, LF mNLC is used to treat Alzheimer's disease [98].

In male Wister rats, Etman et al. designed a microemulsion to deliver piperine to the CNS for the treatment of AD Frequency modulation in the production of the amyloid precursor protein, which decreases A generation, decreases secretase rates, and increases A degradation and transportation out from the brain would be the mechanism for anti-AD therapy. Furthermore, its antioxidant and anti-apoptotic properties contribute to the observed neuroprotection, preventing earlier damage in the pathogenesis of AD [136].

In a clinical sick person having AD, Shadab Mb et al. used in vitro nano-emulsion based naringenin. Even though naringenin can only pass bio-membranes to a minor extent, this is being researched as a flavonoid that might defend neurons from free radicals and inflammation by inhibiting APP and secretase activity, lowering amyloidogenesis in SH-SY5Y molecules and inhibiting hyperphosphorylated tau. Naringenin NEs were observed to get a much stronger neuroprotective effect in vitro than free naringenin against amyloid plaque neurotoxicity. These findings suggest that NEs loaded with naringenin are used to treat AD [125].

Thus, therapeutic compounds such as SLNs of quercetin, piperine solid lipid nanoparticles ApoE, α-mangostin, lactoferrin-conjugated NLC, and Ach has proved to be effective in the in vitro and in vivo models. Further clinical trials are required for the efficient and practical usage of these therapeutic compounds upon delivery.

Currently, a number of non-invasive image-guided modalities, such as MRI, CT, PET, SPECT, electron microscopy, autoradiography, optical imaging, and US, have been used in biomedical and clinic settings. While CT and MRI are frequently used for anatomical imaging, PET and optical imaging are quantitative or semiquantitative imaging modalities [145]. Especially in CMT- and RMT-mediated drug delivery and endocytosis, nanocarriers with appropriate surface characterization or conjugated with various types of ligands are a promising “Trojan horse” approach for the release of therapeutics into tissues and neuronal cells. The idea of ‘theragnostic’ (diagnosis with therapy) can help with the delivery of medications into the CNS and across the BBB in a more precise and direct way [146]. To deliver drugs and diagnostic molecules to the brain across the BBB, drug delivery image guided by NPs with high specificity and multifunctionality can be used. Due to altered pharmacokinetics and biodistribution of the drugs as a result of nanotechnology, these nanoscaled carriers can also reduce the exposure of healthy tissues to drugs [147]. An intriguing new field that can provide real-time tracking of those nanocarriers is direct in vivo imaging of nanomaterials [148].

The BBB can be successfully crossed with intranasal drug delivery, including peptide delivery. For instance, an intranasal administration combined with nanotechnology was studied by Wen et al., to increase the target effect on the brain. Poly (ethylene glycol)-poly (lactic-*co*-glycolic acid) (PEG-PLGA) nanoparticles were coated with the odorranalectin (OL) lectin for nasal administration. Intranasal administration had better brain targeting effectiveness than intravenous injection, according to fluorescence imaging [149].

In a study by Kumar et al., risperidone was delivered to the brain using a nanoemulsion technique. The brain: blood uptake ratio in the rat brain after nasal administration was higher than that attained by intravenous injection, according to in vivo gamma scintigraphy [150]. In previous studies, it has been demonstrated that beta-amyloid and tau pathology are reduced in animal models of Alzheimer's disease when combined with magnetic resonance-guided focused ultrasound and intravenously injected microbubbles [151]. A study conducted by Lipsman et al.*,* showed that in the phase I safety trial, five patients with early to moderate Alzheimer's disease had the blood-brain barrier broken by focused ultrasound. According to the findings, there were no significant radiographic or clinical adverse events, nor did cognitive scores at three months worsen relative to baseline. To confirm amyloid deposition at the target site, beta-amyloid levels were assessed prior to treatment using [^18^F]-florbetaben PET. No group-wise changes in amyloid post-sonication were indicated by exploratory analysis. As a potential novel treatment and delivery method for patients with Alzheimer's disease, focused ultrasound is still being investigated, according to the findings of this safety and feasibility study [152].

Based primarily on the indirect tracking of morphological changes, X-ray CT has emerged as a significant imaging modality for imaging pharmacokinetics and treatment monitoring. For instance, using a polymer coated Bi_2_S_3_ NP formulation as an injectable CT imaging agent, Rabin et al., reported improved in vivo imaging of the vasculature, the liver, and the lymph nodes in mice model [153]. In a rabbit radiofrequency (RF) ablation model, Maier-Hauff et al., used CT to noninvasively monitor the local drug release [154]. CT is common in clinical settings and that micro-CT and hybrid systems, which combine X-ray CT with PET and SPECT, are becoming more popular, the use of *N*P-based imaging probes for X-ray CT imaging has the potential to have a significant impact on health care. The majority of CT contrast agents are small, iodinated molecules, but due to their non-specific distribution, rapid pharmacokinetics, and low sensitivity, they have had a limited ability to target. Oncological imaging now uses MRI as a tool. Gadolinium, a paramagnetic contrast agent, or superparamagnetic iron oxide nanoparticles (SPIONs) have all been identified as key players in the tracking of single or clusters of labelled cells within target tissues [155].

The simplicity, low cost, and small size of the equipment make imaging with light particularly advantageous. In contrast to other modalities, visible and near-infrared wavelengths offer a wide range of probing mechanisms and highly targeted contrast techniques. Due to the different light absorption spectra of soft tissues, optical contrast techniques have the potential to distinguish between them [156]. Extrinsically administered “switchable” and “tumor-selective” fluorescent optical agents expand the range of potential applications. A wide range of fluorescent probes and markers that are commercially available have become more prevalent over the past ten years, ranging from non-specific fluorescent dyes and fluorescent proteins to targeted or activatable photoproteins and fluorogenic-substrate-sensitive fluorochromes [157]. There have also been developed multifunctional nanoplatforms for both targeted treatment and diagnostics based on protein cage architectures loaded with imaging agents. The visualization of the biodistribution of substances labelled with positron emitters is made possible by positron emission tomography (PET), another non-invasive imaging technology. Due to its high sensitivity, PET has some advantages over CT and MRI [158]. PET and SPECT equipment have a limited range of applications due to their high costs and other issues. Additionally, the images produced by these techniques have poor spatial resolution, making it challenging to accurately identify uptake regions. There is an ongoing search for fresh methods for in vivo visualization of brain-targeting nanocarriers, such as strategies based on sensitive and focused optical contrast [159].

### 6.3 Nano-drug delivery system

#### 6.3.1 Exosomes

AD is significantly influenced by exosomes. They are specifically linked to the mechanisms that produce, remove, and accumulate amyloid-beta (Aβ), which are defining characteristics of AD and the development of neurofibrillary tangles [160,161]. Exosomes are also linked to neuroinflammation, oxidative stress, and tau protein's aberrant phosphorylation, all of which are involved in the generation of Aβ [162]. In order to treat AD by focusing on these pathogenic pathways, exosomes may one day be used as therapeutic agents (Table 4)**.**

**Table 4:** Targeted protein of several drugs, their therapeutic mechanism and pre-clinical study reports.

Sl.No.	Drugs	Targeted Protein	Therapeutic mechanism	Pre-clinical study	Ref.
1	Cur@*Exo*-LFA-1	Tau	Curcumin inhibits the formation of Aβ aggregates	In vivo (C57BL/6 mice, peritoneal injection) synonyms, In vitro (RAW 264.7 ​cells, hCMEC/D3)	[160]
2	3D-Exo	Aβ	3D-Exo activates alpha-secretase and inhibits beta-secretase, resulting in decreased Aβ production.	In vivo (APP/PS1 transgenic mice, Intravenous injection) In vitro (SH-SH5Y-APPswe cells)	[163]
3	MSC-Exo-RVG	Aβ	MSC-Exo-RVG reduces plaque deposition and lowers Aβ levels, resulting in the suppression of astrocyte activation.	In vivo (APP/PS1 double transgenic mice, intravenous injection)	[164]
4	HBMVECs-Exo	Aβ, *P*-gp	HBMVECs-Ex facilitates the effective removal of Aβ from the brain by efficiently transporting Aβ out of the cerebral region	In vivo (C57BL/6 mice, hippocampal injection) In vitro (HBMVECs)	[165]
5	oAβ- Exo	Aβ	oAβ-Exo blocks the biosynthesis, release, or absorption of exosomes	In vitro (AF22 ​cells, SH-SY5Y cells)	[166]
6	p25@AAV9-GFP	Aβ,Tau	p25 suppresses Cdk5/p25 function	In vivo (p25Tg mice, intracerebroventricular injection)	[167]
7	CIP@AAV9	Aβ	CIP inhibits the activity of Cdk5	In vivo (p25Tg mice, intracerebroventricular injection	[168]
8	CB@RBCMs	Aβ	CB blocks cyclooxygenase-2 function	In vivo (APP/PS1 Tg mice) In vitro (SH-SY5Y cells)	[169]
9	CIP@AAV9-GFP	Aβ, Tau	CIP blocks Cdk5 function.	In vivo (male APP/PS1 mice, male C57BL/6 ​J mice, intracerebroventricular injection) In vitro (HEK293 ​cells)	[170]
10	pTau422@HBc-S VLPs	Aβ	pTau422@HBc-S VLPs trigger high antibody titres against pathological pTau422, improve cognitive dysfunction, and decrease phosphorylated tau accumulation and gliosis.	In vivo (BALB/c female mice, APP/PS1 transgenic mice)	[171]
11	*P*C-Fe3O4 NPs	Tau	Fe3O4 suppresses Tau protein aggregation	In vitro (N2a cells)	[172]
12	Cur@RPC NPs-T807/TPP	Tau, Aβ	Curcumin decreases oxidative pressure, enhances neuronal firing, inhibits Aβ clumping, and diminishes phosphorylated tau levels.	In vivo (Male ICR mice, SD rates, intravenous) In vitro (BMECs cells, RAW264.7 ​cells, HT22 ​cells)	[173]

Sinha et al. revealed that exosomes, which are small extracellular vesicles derived from the brains of AD patients, have the ability to increase the levels of amyloid-beta (Aβ) oligomers. These exosomes act as carriers and facilitate the transfer of toxic substances between neurons in culture. Significantly, blocking the formation, secretion, or uptake of exosomes resulted in a decrease in the spread of oligomers and the associated toxicity. These findings suggest that exosomes play a crucial role in the transmission of harmful molecules between cells in AD, highlighting their potential as a target for therapeutic interventions [166].

Losurdo et al. utilized 3xTg mice to investigate the potential of exosomes derived from cytokine-preconditioned mesenchymal stem cells (MSCs) to induce immunomodulatory and neuroprotective effects in AD [174]. Yang et al. used a 2D graphene sheet and a 3D graphene scaffold as a matrix for growing human umbilical cord mesenchymal stem cells in their investigation. From the supernatants of these cell cultures, exosomes were recovered. It was discovered that these exosomes, known as 3D exosomes, contained a particular cargo that could up-regulate the expression of a-secretase while decreasing the expression of b-secretase [163]. Both AD pathology cells and transgenic animals produced less amyloid-beta (Aβ) due to this molecular modification. The results point to a promising direction for AD treatment research in that 3D exosomes created from the graphene scaffold-based culture system may be able to control Aβ synthesis [165].

#### 6.3.2 VLPs

Reactive amino acids like lysine and glutamic acid are present on the vast surfaces of VLPs. Clinical experiments have employed a particular VLP known as HBc-*S*-p tau422 [175]. High antibody titers can be produced by this VLP against tau422 that has been pathologically phosphorylated, a protein linked to AD. A further promising method for treating cognitive impairments in APP/PS1 transgenic mice is the injection of HBc-*S*-p tau422 [176] **.** Overall, VLPs, especially HBc-*S*-p tau422, show promise as a treatment for AD due to their capacity to stimulate an immune response against tau422 and enhance cognitive performance [176]**.** Garca et al. examined a number of studies concentrating on the creation of novel (AD) vaccine candidates. These particles were created using modified vaccinia Ankara (MVA) vectors that expressed either the full-length version of the human tau 4R2N isoform or the mutant tau 3 ​R ​C protein (referred to as MVA-tau3RC) [177].

CIP@AAV9-GFP is a vector that He et al. created to deliver a Cdk5 inhibitory peptide (CIP) using an adeno-associated virus serotype 9 (AAV9) [170]. In both in vivo and in vitro tests, their findings showed that CIP@AAV9-GFP had advantageous effects. It was discovered to lessen apoptosis, amyloid-beta (Aβ) aggregation, microgliosis, and hyperphosphorylation of tau protein. Additionally, CIP@AAV9-GFP was effective in regaining cognitive function. Two more studies, in addition to He et al. used CIP@AAV9 in various mouse models, one in an adult mouse model overexpressing p25 and the other in a p25Tg mouse model [167]. Both investigations found that CIP@AAV9 might lessen the neurotoxicity brought on by p25, despite differences in the AD mice models that were utilized.

#### 6.3.3 Cell membrane coating particles

Cell membrane coating technology imitates the characteristics of cell membranes for a variety of uses. This strategy is the subject of ongoing research and has enormous potential for nanoscale biomedicine [178,179]. It is feasible to integrate the natural features of the cell membrane with those of the synthetic inner core material by coating nanoparticles (NPs) with cell membranes. Targeted functions are made possible by the coating's improvement of the NPs' biocompatibility, efficacy, and circulation inside the body [179]. The RBC membrane is one of the various cell membrane types that is frequently utilized in AD therapy. RBC membrane is a frequently used option in AD therapy. Overall, cell membrane coating technology shows promise for the development of improved therapeutics and targeted drug delivery systems in biomedical applications [180].

Guo et al. developed a delivery system called CB@RBCMs, which involved coating nanoparticles with erythrocyte membranes (RBCM) to deliver the specific COX-2 inhibitor celecoxib (CB). This approach proved effective in mitigating neurotoxicity induced by lipopolysaccharide or amyloid beta (Aβ). CB@RBCMs exhibited sustained release of CB over 72 ​h in vitro and demonstrated high biodistribution efficiency in the brain after intranasal administration. Consequently, they were successful in clearing aggregated Aβ in neurons [181]. Further, Gao et al. developed a method to deliver Curcumin (Cur) to neuronal mitochondria in a different investigation. They added antioxidants to human serum albumin nanoparticles (RPC NPs) that have T807 and TPP molecules attached to the surface of the RBC membrane to disguise it (Cur@RPC NPs-T807/TPP). This strategy enabled neuronal targeting, blood-brain barrier (BBB) penetration, and subsequent localisation in the mitochondria. Results from in vitro and in vivo experiments showed that the Cur-loaded T807/TPP-RBC-NPs significantly decreased the signs and symptoms of Alzheimer's disease (AD) by reducing mitochondrial oxidative stress and preventing neuronal death. Overall, the studies showed the potential of erythrocyte membrane-coated nanoparticles as efficient delivery systems for medicinal substances like celecoxib and curcumin in preventing neurotoxicity, lowering amyloid-beta levels, relieving mitochondrial oxidative stress, and preventing neuronal death in the context of Alzheimer's disease.

## 7 Conclusion and future perspective

This review gives an insight into the use of lipid nanosystems as a paradigm for delivering therapeutic compounds for AD. The molecular pathogenesis of AD has been discussed. The challenges posed by the BBB indirect drug delivery have been discussed in this review. Lipid nanosystems have emerged as a promising therapeutic approach for treating Alzheimer's disease. Alzheimer's disease is a neurodegenerative disorder that affects millions of people worldwide. Despite extensive research efforts, there is no cure for Alzheimer's disease. However, using lipid nanosystems as a drug delivery system has shown great potential in treating Alzheimer's disease by enhancing drug efficacy and reducing toxicity. In this conclusion and future perspective, we will focus on the clinical application of lipid nanosystems for Alzheimer's disease therapy. Lipid Nanosystems, such as liposomes, solid lipid nanoparticles, polymeric nanoparticles, nanostructured lipid carriers, and nanoemulsions, have been extensively studied for their potential use in treating Alzheimer's disease. These nanosystems offer several advantages, such as biocompatibility, stability, and the ability to encapsulate hydrophilic and hydrophobic drugs. Moreover, lipid nanosystems can effectively deliver drugs to the brain, which is a major challenge in treating Alzheimer's disease due to the blood-brain barrier. Preclinical studies have shown promising results for using lipid nanosystems in treating Alzheimer's disease. While preclinical studies have shown promising results, the clinical application of lipid nanosystems for Alzheimer's disease therapy is still in its early stages. However, several clinical trials are currently underway investigating the use of lipid nanosystems for Alzheimer's disease therapy. For example, a phase II clinical trial is underway to investigate liposomal alendronate, a bisphosphonate drug's safety, and efficacy in treating Alzheimer's disease (NCT02609152). Several challenges must be addressed to effectively translate lipid nanosystems from preclinical studies to clinical applications. One of the major challenges is the scalability and reproducibility of lipid nanosystems. Producing lipid nanosystems in large quantities and with consistent quality is critical for clinical application. Additionally, the safety and toxicity of lipid nanosystems need to be carefully evaluated to ensure their clinical safety. In the future, the development of lipid nanosystems for Alzheimer's disease therapy will depend on several factors, such as advances in drug discovery and development, improvements in nanotechnology, and the availability of funding and resources. Moreover, lipid nanosystems may be combined with other therapeutic approaches, such as gene therapy and immunotherapy, to enhance their efficacy and provide a more comprehensive treatment for Alzheimer's. In conclusion, lipid nanosystems have shown great potential in treating Alzheimer's disease by enhancing drug efficacy and reducing toxicity. While preclinical studies have shown promising results, the clinical application of lipid nanosystems for Alzheimer's disease therapy is still in its early stages. However, ongoing clinical trials, drug discovery and development advances, and nanotechnology hold promise for the future of lipid nanosystems in treating Alzheimer's disease.

## Declaration of competing interest

The authors declare that they have no known competing financial interests or personal relationships that could have appeared to influence the work reported in this paper.

## Data Availability

Data will be made available on request.
